# SARS-CoV-2 Infection in Health Care Personnel and Their Household Contacts at a Tertiary Academic Medical Center: Protocol for a Longitudinal Cohort Study

**DOI:** 10.2196/25410

**Published:** 2021-04-30

**Authors:** Emily J Ciccone, Paul N Zivich, Evans K Lodge, Deanna Zhu, Elle Law, Elyse Miller, Jasmine L Taylor, Suemin Chung, Jason Xu, Alexander Volfovsky, Cherese Beatty, Haley Abernathy, Elise King, Haley E Garrett, Alena J Markmann, Meghan E Rebuli, Subhashini Sellers, David J Weber, Raquel Reyes, Naseem Alavian, Jonathan J Juliano, Ross M Boyce, Allison E Aiello

**Affiliations:** 1 Division of Infectious Diseases School of Medicine University of North Carolina Chapel Hill, NC United States; 2 Department of Epidemiology Gillings School of Global Public Health University of North Carolina Chapel Hill, NC United States; 3 Carolina Population Center University of North Carolina Chapel Hill, NC United States; 4 School of Medicine University of North Carolina Chapel Hill, NC United States; 5 Institute for Global Health and Infectious Diseases University of North Carolina Chapel Hill, NC United States; 6 Department of Statistical Science Duke University Durham, NC United States; 7 Department of Pediatrics, Center for Environmental Medicine, Asthma and Lung Biology School of Medicine University of North Carolina Chapel Hill, NC United States; 8 Division of Pulmonary Diseases and Critical Care Medicine School of Medicine University of North Carolina Chapel Hill, NC United States; 9 Division of Hospital Medicine School of Medicine University of North Carolina Chapel Hill, NC United States; 10 Curriculum in Genetics and Molecular Biology School of Medicine University of North Carolina Chapel Hill, NC United States

**Keywords:** SARS-CoV-2, COVID-19, health personnel, cohort studies, Bluetooth contact tracking, survey-based research, occupational health, seroprevalence, mobile phone

## Abstract

**Background:**

Health care personnel (HCP) are at high risk for exposure to the SARS-CoV-2 virus. While personal protective equipment (PPE) may mitigate this risk, prospective data collection on its use and other risk factors for seroconversion in this population is needed.

**Objective:**

The primary objectives of this study are to (1) determine the incidence of, and risk factors for, SARS-CoV-2 infection among HCP at a tertiary care medical center and (2) actively monitor PPE use, interactions between study participants via electronic sensors, secondary cases in households, and participant mental health and well-being.

**Methods:**

To achieve these objectives, we designed a prospective, observational study of SARS-CoV-2 infection among HCP and their household contacts at an academic tertiary care medical center in North Carolina, USA. Enrolled HCP completed frequent surveys on symptoms and work activities and provided serum and nasal samples for SARS-CoV-2 testing every 2 weeks. Additionally, interactions between participants and their movement within the clinical environment were captured with a smartphone app and Bluetooth sensors. Finally, a subset of participants’ households was randomly selected every 2 weeks for further investigation, and enrolled households provided serum and nasal samples via at-home collection kits.

**Results:**

As of December 31, 2020, 211 HCP and 53 household participants have been enrolled. Recruitment and follow-up are ongoing and expected to continue through September 2021.

**Conclusions:**

Much remains to be learned regarding the risk of SARS-CoV-2 infection among HCP and their household contacts. Through the use of a multifaceted prospective study design and a well-characterized cohort, we will collect critical information regarding SARS-CoV-2 transmission risks in the health care setting and its linkage to the community.

**International Registered Report Identifier (IRRID):**

DERR1-10.2196/25410

## Introduction

### Background

As of October 2020, the global COVID-19 pandemic accounts for more than 43 million confirmed infections and 1.1 million deaths, along with unprecedented disruption to social networks and economic systems [1]. The etiologic agent, the SARS-CoV-2 betacoronavirus, is primarily spread from person to person via inhalation or direct contact with aerosolized droplets. Frontline health care personnel (HCP) have been shown to be at increased risk of infection due to frequent exposure to, and close contact with, infected patients and contaminated surfaces [2-4]. Shortages of critical personal protective equipment (PPE) during the pandemic have further exacerbated risks to HCP. A review of the epidemiological data from the site of the initial SARS-CoV-2 outbreak in Wuhan, China, showed that 62.9% (1080/1716) of HCP were infected with SARS-CoV-2, and 14.8% (247/1668) of HCP developed severe disease [5]. The seroprevalence of SARS-CoV-2 among HCP in the United States and Europe appears to be far lower, ranging from 1.9% to 12.6%, with 0.0% to 1.7% of those infected being hospitalized due to severe disease [6]. Data from two nosocomial outbreaks of SARS-CoV-2 in the United States demonstrate that the risk of virus transmission to HCP is highest during episodes of close patient contact without adequate PPE [7].

Infected HCP may also contribute to disease transmission, both in the hospital setting and in the community. When infection results in overt clinical symptoms, identifying and isolating infected HCP is relatively straightforward. However, many SARS-CoV-2–infected individuals are asymptomatic or develop only mild symptoms [8-10]. In the absence of regular screening, asymptomatic individuals are unlikely to isolate or seek care. Yet, even asymptomatic or presymptomatic individuals can harbor high viral loads in respiratory secretions and may account for high numbers of secondary infections [8,9]. Outside of the hospital, little is known about the role HCP play in the transmission of SARS-CoV-2, particularly among household members and close contacts. However, numerous studies have demonstrated that the household is an important venue for SARS-CoV-2 transmission [11]. Potential transmission from HCP to family members and other close contacts is a source of considerable stress that may adversely impact mental health and job performance [12].

Most studies of SARS-CoV-2 seroprevalence among HCP have been cross-sectional or case series analyses [13]. Although basic longitudinal analyses of SARS-CoV-2 incidence among HCP in the United States have also been conducted, none have included household contacts and none have digitally tracked HCP interactions and movement in the health care setting to identify potential points of risk and transmission [14]. Therefore, there is a need to better study the prospective risk factors for SARS-CoV-2 infection among HCP, track their interactions in the workplace, and quantify infection risks among their household contacts.

### Study Objectives

The overarching goal of the study was to quantify and describe the risk of SARS-CoV-2 infection among frontline HCP, ancillary support staff, and their household contacts amidst the ongoing COVID-19 pandemic. To accomplish this goal, we designed a layered prospective cohort study of HCP and their household contacts. We enrolled frontline HCP, including physicians, nurses, and ancillary staff, providing and supporting emergency room, respiratory diagnostic testing, and inpatient care at a large, academic, tertiary care medical center. We collected survey data, venous blood samples, and a nasal swab for detection of SARS-CoV-2 every 2 weeks for 3 months and then monthly thereafter for a period of up to 6 months. We also tracked individuals’ interactions using Bluetooth sensors that were linked to a smartphone app for each participant and also attached to various locations within their workplace. Our central hypothesis was that preventive behaviors outside of work, interactions in the workplace, and use of protective equipment would predict the risk of infection in HCP and their household contacts.

## Methods

### Study Design Overview

**Figure 1:**
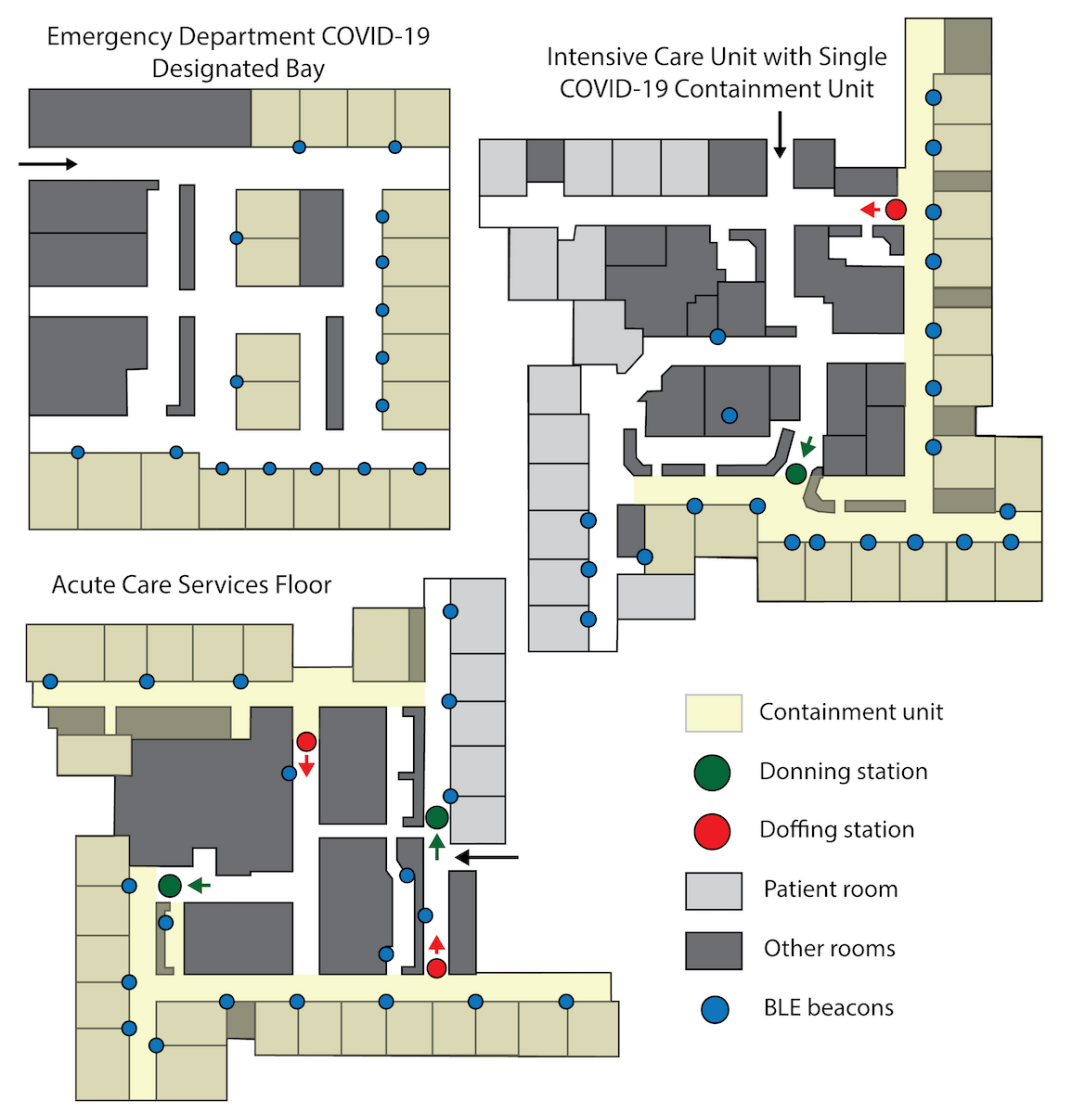
Schematic representation of hospital units in which patients with COVID-19 are cohorted with locations of environmental Bluetooth Low Energy (BLE) beacons.

We conducted a prospective, observational study of SARS-CoV-2 among HCP and their household contacts during a global pandemic at a large, regional, southern US medical center. The setting was a tertiary care facility with over 900 beds. The SARS-CoV-2 response at the medical center involved the cohorting of suspected and infected patients on particular floors (see Figure 1) and localization of care by specific teams of providers. Within the hospital, this included a team in the intensive care unit and on a medical floor, staffed by a subset of medical providers (ie, physicians, advanced practice providers, respiratory therapists, and nurses) as well as ancillary staff (ie, environmental services, food services, and rehabilitation therapists). Both the medical providers and ancillary staff at times also worked on other floors in the hospital. The Emergency Department (ED) was responsible for evaluating and admitting the most severe infections. Together, these providers were at the highest risk for SARS-CoV-2 exposure. Outside the hospital, the medical center was operating a drive-through testing center to allow for the diagnosis of SARS-CoV-2 in ambulatory patients away from the main hospital. Providers and support personnel at the drive-through site routinely interacted with patients before knowing the individual’s infection status.

Based on sample size calculations (see Data Analysis section), we sought to enroll 300 HCP providing care and services in inpatient, ED, and testing center settings during the COVID-19 pandemic. Of note, HCP were not routinely screened for SARS-CoV-2 infection at this institution. Eligibility criteria for HCP included the following: (1) provided patient care or support services at the University of North Carolina Medical Center (UNCMC) or the Respiratory Diagnostic Center (RDC) during the COVID-19 pandemic, (2) planned to remain employed by the University of North Carolina (UNC) for the duration of the study, (3) willing and able to provide informed consent or assent, and (4) had access to stable internet, email, and a computer at home. The only exclusion criterion was an inability to provide informed consent. As described in detail below, HCP and ancillary staff were surveyed regarding potential occupational exposures and patient care activities. Venous blood, nasal swabs, and nasal epithelial lining fluid (NELF) were collected every 2 weeks. HCP participants who developed clinical symptoms at any point during the study and were not self-identified as SARS-CoV-2 positive were referred to Occupational Health for evaluation. Additionally, proximity contacts between individual HCP participants and between HCP participants and high-risk locations in the hospital were collected via a mobile app using Bluetooth technology.

Finally, we enrolled a subset of the household members of HCP participants to assess transmission dynamics outside of the hospital environment. For household members to be considered eligible, they had to (1) cohabitate for an average of at least 40 hours per week with an HCP participant, (2) have the ability to use a collection device for serum collection on one’s own or with assistance from another household member, and (3) be willing and able to provide informed consent or assent or parental consent. Those younger than 18 months of age and those who were unable to provide informed consent, or parental consent if under 18 years of age, were excluded from the household study. This study was approved by the Institutional Review Board of the UNC at Chapel Hill (20-0942).

### Recruitment and Consent Process

#### Health Care Personnel

To recruit HCP, study leaders met with administrative leadership for divisions within the Department of Medicine (Infectious Diseases, Pulmonary and Critical Care, Hospital Medicine, etc) and other departments within the School of Medicine (Pediatrics, ED, etc), nursing leadership for hospital units, respiratory therapy administrators, and leadership for ancillary and support services (eg, environmental health, patient transport, and food services) to present and discuss the study. Various additional approaches for recruitment were used as well, including (1) direct communication to personnel through email or face-to-face encounters; (2) presentation or discussion about the study at virtual or in-person staff meetings; (3) flyers, posters, or other public displays; and (4) small gifts on which the study logo was printed, such as miniature hand sanitizer bottles, pens, and stress balls. Interested individuals were referred to a study-specific website where they were able to learn more about the study, ask questions through a study-specific email address, and submit an online prescreening survey to communicate their interest to the study team. The study team then scheduled a time to discuss the study with participants by phone and complete an electronic consent form, a copy of which was emailed to the participant. HCP participants were able to opt out of electronic contact tracking and collection of NELF samples on the consent form but still participate in the other study procedures. Once enrolled, a unique participant identifier was generated for linkage of data sources. HCP recruitment and enrollment began in July 2020 and is ongoing as of January 2021.

#### Households

Due to logistical and financial constraints, we could not include all households of participating HCP in the study. Therefore, to recruit household members of HCP participants, we selected a random sample of high- and low-risk HCP participants for household investigation every 2 weeks. HCP were considered high risk if any of the following occurred:

1. HCP self-reported contact with suspected or confirmed patient with SARS-CoV-2 infection without proper PPE, defined as N95 respirator, gown, gloves, and eye protection.
2. HCP self-reported errors and/or malfunction of PPE during patient encounters.
3. Positive SARS-CoV-2 rapid serology test result: either immunoglobulin (Ig) M or IgG.
4. Suspected or confirmed SARS-CoV-2 infection.

If none of the above criteria applied, the HCP was considered low risk. Once participants were stratified into high- and low-risk categories, a third-party biostatistician performed probability-based sampling to select 6 to 10 households for investigation (3 to 5 households per risk category). Study staff were masked to the assigned risk categories for the selected households. The HCP were approached to provide emails and ages for all household contacts. If the HCP participant agreed to the household investigation, the study team contacted their household members about study participation by phone. The study team reviewed the study documents, electronic consent form, and the process for at-home sample collection with the household member. If the household member agreed to participate, they signed the consent form electronically. Each household member was provided a handout about when to seek care for potential SARS-CoV-2 infection and when to contact the study staff by email. Household member recruitment began in July 2020 and is ongoing as of January 2021.

### Study Procedures for Health Care Personnel

**Figure 2:**
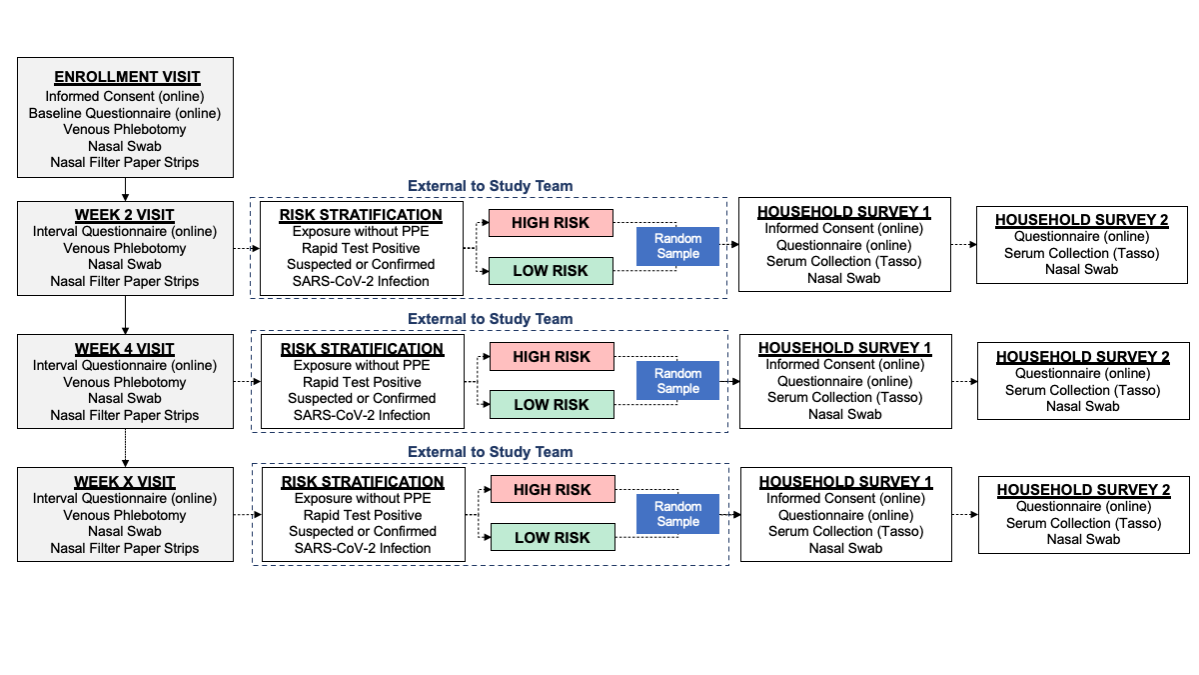
Overview of the study design including health care personnel participant visits and household selection process. PPE: personal protective equipment.

The overview of the study design is shown in Figure 2. Questionnaires for HCP used in the study can be found in Multimedia Appendices 1-4.

#### Baseline Survey

At baseline, HCP participants received an electronic questionnaire regarding demographics, occupational duties, current symptoms, known exposures to SARS-CoV-2-infected persons, and PPE usage. This initial questionnaire also gathered data about the participant’s household (eg, number of people and occupations) and included basic mental health assessments for depression, anxiety, and posttraumatic stress disorder (PTSD).

#### Daily Surveys

Once the first in-person study visit was completed (see Study Visits and Sample Collection section below), daily electronic surveys were sent to participants for the first 12 weeks of study participation via an automated email reminder. Questions included a clinical symptom assessment, with self-recorded temperature if symptomatic; hours worked at the medical center; number of contacts with patients with suspected or confirmed SARS-CoV-2; and occupational activities performed during interactions with patients.

#### Biweekly Surveys

The participant received a longer electronic survey by email every 2 weeks containing questions concerning current symptoms, known exposures to SARS-CoV-2-infected individuals, PPE availability and usage since they completed the last 2-week questionnaire, and pandemic-related stress.

Throughout the study, if a participant indicated symptoms consistent with SARS-CoV-2 infection, they were referred to the medical center’s Occupational Health Department for evaluation and testing. Additionally, if a participant indicated that they were having thoughts of self-harm more than half of the days in the last 2 weeks, they were sent an email referral to an institutional mental health hotline that was developed to address the mental health support of all HCP as well as other local and national mental health support resources.

#### Study Visits and Sample Collection

##### Baseline

After completion of the baseline questionnaire, a research assistant met the participant at one of three designated sites in or near the medical center for the baseline study visit, during which the following study procedures took place:

1. Venous blood was collected in an EDTA tube by trained phlebotomists for SARS-CoV-2 IgM/IgG rapid diagnostic testing and preparation of dried blood spots (DBS) for storage. Venous blood was also collected in two 8 mL cell separator tubes for peripheral blood mononuclear cell (PBMC) isolation and storage, using the BD Vacutainer Mononuclear Cell Preparation Tube (CPT), Sodium Citrate (BD Biosciences). Plasma was isolated from the cell preparation tubes for SARS-CoV-2 antibody testing by enzyme-linked immunosorbent assay (ELISA).
2. We asked a subset of participating HCP (100 individuals) to have blood drawn for SARS-CoV-2 antibody testing by ELISA using the Tasso serum self-collection kit (Tasso, Inc) in parallel with venous phlebotomy as part of a substudy. Tasso devices were used per the manufacturer’s instructions. Briefly, individuals washed their hands and prepped their skin by rubbing until warm and wiping with alcohol to sterilize. The device was then adhered to the skin for 5 minutes to collect the sample and removed.
3. Participants self-collected a mid-turbinate nasal swab (MTNS) using the protocol described in previous studies [15,16]. We employed MTNS as opposed to nasopharyngeal swabs as this method of collection has demonstrated comparable performance for the identification of respiratory pathogens [12], while involving less risk to both the subject and study staff. The swab was placed in a collection tube filled with DNA/RNA Shield (Zymo Research) for SARS-CoV-2 polymerase chain reaction (PCR) testing.
4. Participants self-collected NELF using absorbent strips inserted into the nose—if they opted into this part of the study—as described previously (see Multimedia Appendix 5) [17]. Briefly, participants moistened the nasal passage using a metered spray bottle and normal saline. They then inserted absorbent strips, cut to fit into the nasal passages, into each nare. Participants then clamped the strips in place with a padded nasal clamp, to ensure maximal contact with the nasal mucosa, for 2 minutes. Participants then removed the strips from the nose and placed them into provided storage tubes.
5. Each individual was given a digital thermometer to track and report their temperature at home.
6. Participants who opted into downloading an app to track their interactions in the workplace were given a Bluetooth Low Energy (BLE) beacon for contact tracking to attach to their hospital-issued ID badge or lanyard. They were also instructed on how to download and set up the Ethica (Ethica Data) smartphone app (see Network Data Collection section).

##### Follow-up Study Visits

**Figure 3:**
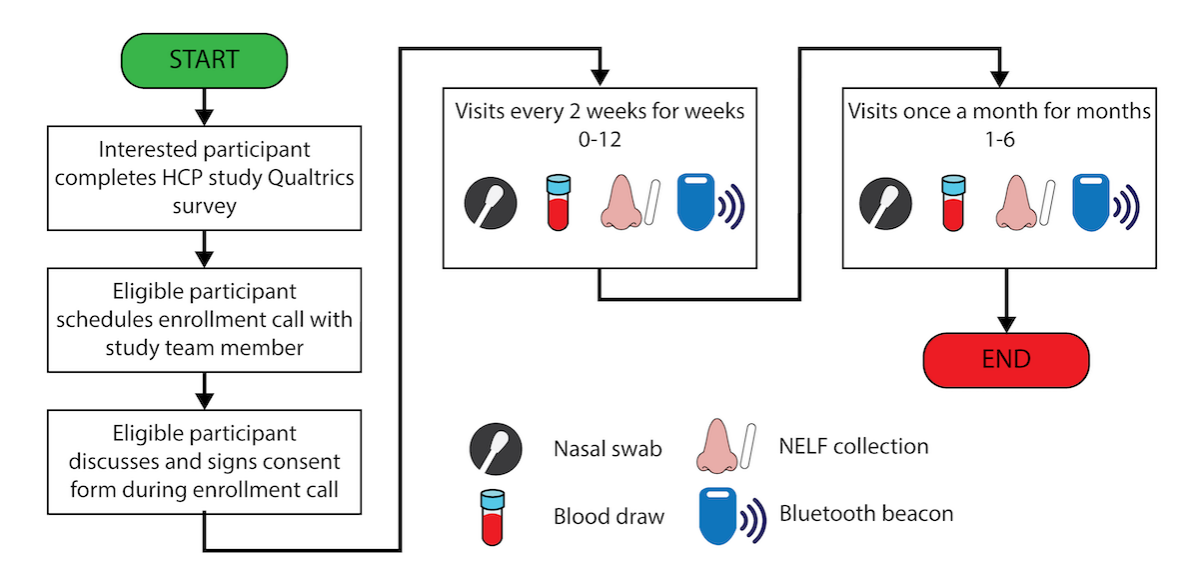
Enrollment and in-person study procedures for health care personnel (HCP) participants. NELF: nasal epithelial lining fluid.

Every 2 weeks for the first 12 weeks and then monthly for the remainder of the study, participants again presented to one of three designated sites for an in-person, follow-up study visit (see Figure 3). Venous blood, MTNS, and NELF strip (if opted in) samples were collected at each visit as above. To minimize potential exposure to study staff, if a participant was due for their biweekly visit but was experiencing symptoms consistent with SARS-CoV-2 infection or had recently tested positive by PCR and was within the quarantine period recommended by Occupational Health, they were not seen in person by the study staff and instead were sent a home collection kit containing a Tasso serum self-collection kit and nasal swab.

### Study Procedures for Households

The overview of the study design is shown in Figure 2. Questionnaires for household members used in the study can be found in Multimedia Appendices 6-8.

#### Baseline Survey

At the time of enrollment, each household member received a survey regarding demographics, relationship to the HCP participant, occupation, medical history, and any recent clinical symptoms, SARS-CoV-2 exposures, or past SARS-CoV-2 testing.

#### Weekly Surveys

Enrolled household members completed weekly questionnaires about possible symptoms of SARS-CoV-2 infection and any testing since the previous survey.

#### Day 21 Survey

At the end of their study participation, 21 days after their first sample collection, household members completed a final questionnaire asking about any changes in occupation since the baseline survey and any SARS-CoV-2 symptoms, exposures, and/or testing since the previous weekly survey.

Throughout their participation in the study, if a household member reported fever and either cough or shortness of breath, they were referred to the testing center or their primary care provider’s office for testing.

#### Sample Collection

Samples were collected at baseline and at day 21. Within 3 days of enrollment, the study team mailed a kit for at-home baseline sample collection to each consented household member; the kit included the following:

1. Tasso at-home serum collection kit for SARS-CoV-2 antibody testing by ELISA. The Tasso device was provided in a box labeled with the date and patient identifier; the box contained written instructions and a link to the website that demonstrates use of the collection device [18]. Once collected, the participant placed the device in a sealable biohazard bag and then into the Tasso kit’s box.
2. Nasal swab and DNA/RNA Shield Collection Tube for MTNS collection for SARS-CoV-2 PCR testing. The nasal swab was provided with an instruction sheet and link to an online video with instructions for nasal swab specimen collection. After specimen collection, the participants placed the sealed tubes containing the swabs in sealable biohazard bags.

Completed at-home collection kits were then packaged in a preaddressed, preposted box and mailed back to the laboratory to minimize face-to-face contact between the study team and household members. The same at-home sample collection process was then repeated 21 days after baseline sample collection.

### Other Study Procedures

#### Network Data Collection

**Figure 4:**
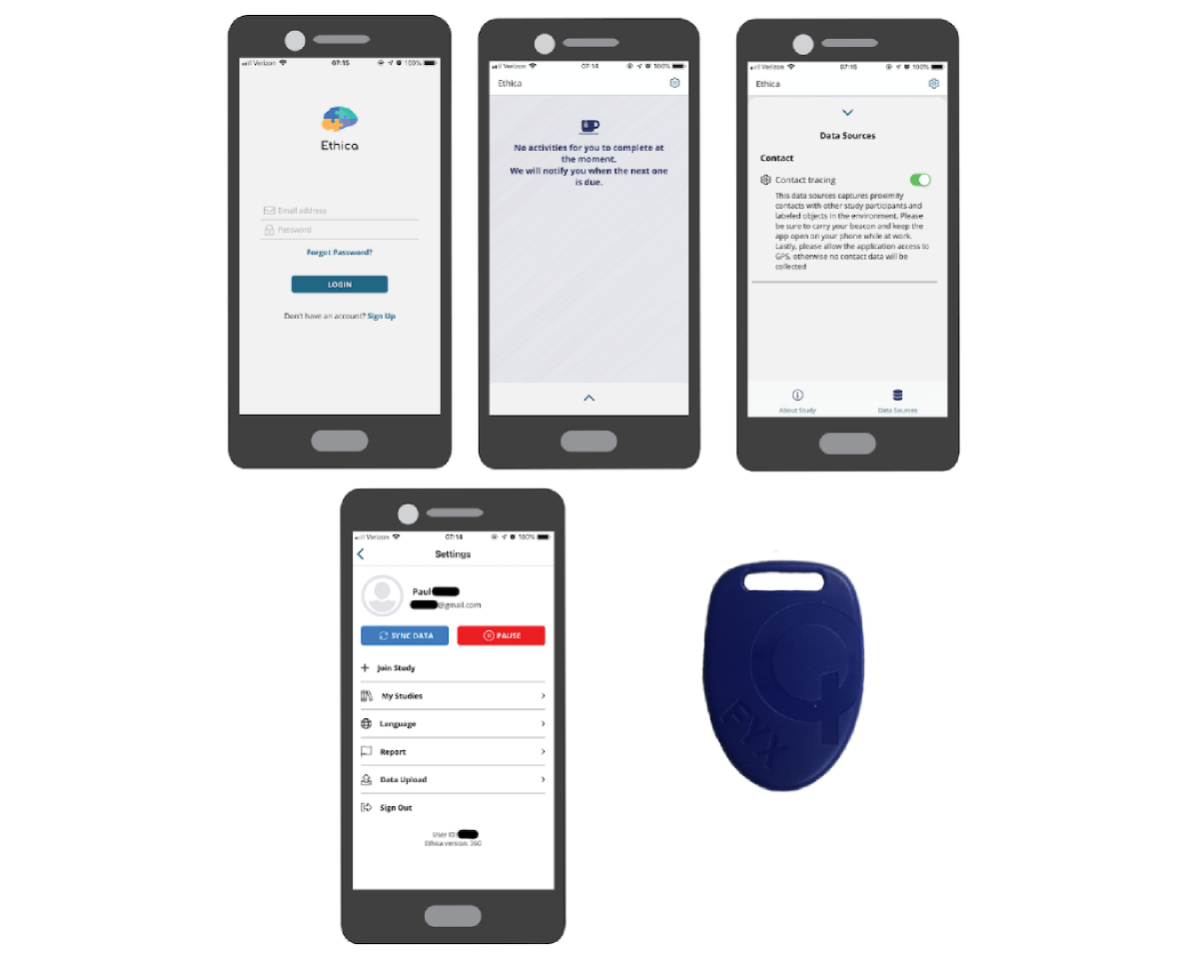
Participant-facing Ethica smartphone app and Bluetooth beacon for collection of proximity contacts. Ethica smartphone app from left to right starting at the top: log-in page, home page for the study, data sources collected and option to pause participation, and settings page. The beacon (shown at the bottom right) dimensions are 1.6 inches (length) by 1.1 inches (width) by 0.2 inches (height).

HCP participant–participant and HCP participant–environment interactions were collected electronically via BLE with the Ethica (Ethica Data) smartphone app (see Figure 4). We followed a similar protocol previously described in our earlier work [19,20]. The Ethica app functions with the use of two devices: the participant’s cell phone and a BLE beacon. While the participant has the Ethica app open on their phone and Bluetooth is active, the app passively collects and records incoming Bluetooth signals with a universally unique identifier (UUID) that corresponds to the study. Due to phone manufacturer constraints, scanning for Bluetooth signals is done at a resolution of approximately 5 minutes. Information recorded for a valid UUID includes the Ethica-assigned identifier for the participant, time of the incoming signal, strength of the incoming signal as measured by the received signal strength indicator (RSSI), and identifiers corresponding to the incoming valid beacon signals. Data are stored locally on the participant’s smartphone until uploaded to the Ethica secure servers through a wireless connection (ie, Wi-Fi or cell signal).

BLE beacons are devices used to broadcast a set of identifiers at a predetermined rate. For our app, BLE beacons were programmed with a unique combination of UUIDs, major values, and minor values as assigned by the Ethica system. While other devices can see the broadcast beacon identifiers, the beacon identifiers are not linked to the other participants’ information or data sources, aside from a linkage within Research Electronic Data Capture (REDCap), the electronic data management system used for the study [21]. BLE beacons can be further designated as participant-owned or environmental. Therefore, proximity to both other participants carrying beacons and beacons placed in the environment can be recorded. For our implementation, Series 10 beacons (Gimbal, Inc) were used. BLE beacons were configured using the iBeacon configuration. Environmental beacons were set at a power level of –8 dBm, which corresponds to an approximate capture distance of 20 feet in internal testing. Participant beacons were set at a power level of –18 dBm, corresponding to 9 feet. To increase the likelihood that a beacon was recorded during the time window of collection by the phone, the beacon transmission was set to 1 Hz. In summary, the BLE beacon broadcasts the participant’s identifier, and the participant’s smartphone collects incoming Bluetooth signals from nearby environmental and participant BLE beacons.

During the consent process, HCP had the option to opt into the proximity contact data collection. HCP who consented to use the Ethica app were given instructions on installing the app on their smartphone. Environmental BLE beacons were placed at locations within the ED, the RDC, the Medical Intensive Care Unit (MICU), pediatric intensive care units, elevators, and floor COVID-19 units for adults and children (see Figure 1). BLE proximity data were downloaded weekly by study staff from the Ethica web portal and monitored.

#### PPE Observations

To document trends in the use of PPE, members of the research team were stationed in the areas in which care for patients with suspected or confirmed SARS-CoV-2 infection most often took place. At least one team member observed each of the following areas once weekly for 2 to 3 hours each time: the ED bay where patients suspected of having SARS-CoV-2 infection were cohorted, the inpatient ward for SARS-CoV-2-infected patients, and the MICU. The observations were collected on an electronic form, including unit-level PPE use and availability and individual HCP-patient interactions; specifically, appropriateness of hand hygiene and PPE use and procedures performed by the provider. This observational data collection will supplement the self-reported daily PPE usage information by the HCP.

### Sample Processing and Testing Procedures

The collection, processing, and storage of samples followed national and international guidelines, and all processes were approved by Environmental Health Services at UNC [22]. Please see Multimedia Appendix 9 for the full laboratory protocol.

#### Venous Blood

##### SARS-CoV-2 IgM/IgG Rapid Diagnostic Test

The rapid diagnostic test (RDT) followed the manufacturer's guidelines (Elabscience) [23]. Briefly, 20 μL of whole blood was applied to the sample well using an enclosed micropipette. Three drops of reagent were added to the sample well. The RDT was read after 10 minutes.

##### Dried Blood Spot Processing

Approximately 200 μL of blood was used to make four DBS per patient time point. Blood was spotted on appropriately labeled Whatman 904 cards. Each card was dried for 15 minutes, or until completely dried, before packaging in individual Ziploc bags with desiccant. Blood spots were stored at –80 °C for future research.

##### ELISA for SARS-CoV-2 Antibody

Using both the plasma collected from HCP participants during their in-person study visits and the household participants’ self-collected serum samples, we performed an ELISA using the recombinant spike protein antigen to detect total SARS-CoV-2 Ig in plasma [24]. The cutoff to differentiate a positive versus negative result for the ELISA assay was chosen to ensure the test had 99.5% specificity per US Centers for Disease Control and Prevention (CDC) recommendations for COVID-19 serology testing [25].

##### Peripheral Blood Mononuclear Cell Isolation

Cell preparation tubes were processed and PBMCs isolated as previously described and as detailed in Multimedia Appendix 9 [26]. PBMCs were stored in aliquots of 3 to 6 million cells/μL at –80 ^°^C for future research.

#### Mid-Turbinate Nasal Swabs

MTNS were used for SARS-CoV-2 PCR testing. The DNA/RNA Shield medium in which the swabs were collected inactivates all viral particles. RNA was extracted from 200 μL of DNA/RNA Shield medium using a Qiagen HT system. Samples were screened for SARS-CoV-2 infection using the Thermo Fisher Scientific COVID-19 multiplex real-time PCR assay including the MS2 phage spike during extraction. All samples that tested positive by this initial test underwent confirmation testing with the CDC assay and viral copies were quantified [27]. PCRs were batched and assayed retrospectively. For clinical diagnosis of symptomatic individuals, participants were referred to Occupational Health for standard PCR testing at a rapid diagnostic testing center.

#### Nasal Epithelial Lining Fluid

Absorbent strips for capturing fluid from the nasal epithelium were frozen at –80 °C for storage and batched for processing. One strip was utilized for protein analysis via multiplex ELISA for cytokines and chemokines, and the second strip was utilized for viral PCR testing. Samples were inactivated prior to analysis via approved protocols, which included heat and chemical treatment. Processing for protein analysis and PCR analysis occurred as described previously [17]. The remaining NELF was stored for other future characterization of the respiratory epithelial immune response and gene expression analysis.

### Data Analysis

All data were stored in a Health Insurance Portability and Accountability Act–compliant database using REDCap [21].

#### Sample Size

Given the rapid emergence and evolving situation regarding the COVID-19 pandemic, limited data were available at the time of study design on which to base sample size estimates. Preliminary data from northern Italy suggested an infection rate approaching 20% or higher [28,29], but this prevalence estimate exceeded what was expected in the southeastern United States in spring 2020 with the implementation of social distancing restrictions and stay-at-home orders. Therefore, a goal sample size of 300 HCP participants and an additional 250 household members was chosen as a logistically feasible enrollment target based on power calculations. The sample size choice was further informed by recent work regarding epidemic spread in dynamic networks as measured via Bluetooth devices, with the selected target sample size exceeding the largest previous such study [19,30].

#### Exposures

Our primary exposure of interest was employment in a department or unit involved in frontline health care of confirmed or suspected SARS-CoV-2-infected patients. Results from this group will be compared to a group of HCP and ancillary staff employed in a department or unit that is not involved in care of patients infected with SARS-CoV-2. Secondary variables of interest also included other occupational factors (eg, type of PPE used and specific occupation) and preventative behaviors outside of work (eg, mask use and handwashing).

#### Outcomes

Our primary outcome of interest was the incidence of SARS-CoV-2 infection defined as either development of SARS-CoV-2-specific antibodies as determined by ELISA (ie, seroconversion) or clinical infection with SARS-CoV-2 confirmed by PCR testing. Secondary outcomes of interest included the following: (1) demographic, clinical, and occupational factors associated with SARS-CoV-2 infection; (2) proportion of confirmed infections that are subclinical and/or asymptomatic; (3) risk of secondary transmission and serial interval within household contacts; (4) analysis of the Bluetooth contact networks to assess the efficacy of community mitigation policies; and (5) agreement between serological testing results obtained from venous blood collection, DBS, and Tasso device. Exploratory outcomes included the characterization of the immune response in the nasal epithelium at various points during SARS-CoV-2 infection and genotypic analyses of the SARS-CoV-2 viral isolates.

#### Confounders

To account for common causes of department employment and SARS-CoV-2 infection, information on demographics (ie, age, race, ethnicity, gender, and household context), occupational factors (ie, PPE use, PPE availability, and occupational characteristics), behaviors outside of work (ie, mask use and hand hygiene), and mental health (ie, symptom screeners for anxiety, depression, and PTSD and stress-level assessments) were collected. Since risk of infection is affected by the characteristics and behaviors of others, we also collected information on locations worked (ie, unit and time spent on the unit) and proximity contacts as measured through the Bluetooth beacons.

#### Proposed Statistical Analyses

Demographic, clinical (ie, baseline medical conditions), and occupational characteristics of the HCP and household member cohorts will be described using standard summary statistics. The risk ratio and risk difference of SARS-CoV-2 infection among HCP participants working in COVID-19 areas versus non-COVID-19 areas, while accounting for identified confounders, will be estimated using Bayesian additive regression trees [31]. We will calculate 95% credible intervals from the posterior. Furthermore, we will explore potential heterogeneity in outcomes as a function of demographic and occupational characteristics. We will analyze household data following methods applied to influenza transmission studies [32,33]. Loss to follow-up has been actively monitored and participants who discontinue the study are asked the reasoning for their discontinued participation. Data managers periodically audit survey responses and participant engagement with the study and reach out directly to those participants who are not submitting data regularly. In addition, we are corroborating SARS-CoV-2 testing information using the medical record. Depending on the extent of missing data and the pattern, we will use multiple imputation or weighting informed by this data. For sensitivity analyses, we plan on evaluating the nonparametric bounds for missing data and other systematic biases [34].

We will determine the test performance (eg, sensitivity and specificity) of the SARS-CoV-2 IgM/IgG RDT using the ELISA antibody test as the reference assay. To validate serological results obtained using the Tasso at-home collection device, we will also assess for concordance between ELISA antibody testing results from samples collected by the Tasso self-collection kit and venous phlebotomy, which is considered the standard specimen-collection procedure, through calculation of the Cohen κ coefficient.

NELF samples will be analyzed using multiplex ELISAs to quantify (1) the expression of cytokines and chemokines important in the upper respiratory tract antiviral immune response and (2) quantitative SARS-CoV-2 viral load. In conjunction with NELF collected from cohorts of patients with SARS-CoV-2 infection from other ongoing research studies, we will evaluate the association of the measured inflammatory mediators and COVID-19 disease severity as defined by the World Health Organization [35]. We will also evaluate the association between epidemiologically observed risk factors for severe COVID-19 disease [36-39] and mediators of antiviral defense.

The information collected from the Bluetooth beacons will be summarized and visualized using network analysis tools, providing insight into the patterns of movement of study participants within the hospital. Because BLE signals can travel through objects (eg, walls and doors), proximity contacts will be filtered by RSSI values determined by internal assessments of the beacons when used in conjunction with the Ethica app. Furthermore, individual-level likelihood-based methods will be used to study the dynamics of epidemic spread as it relates to the network of individual contacts and potential explanatory variables [30].

## Results

Recruitment for this study is currently ongoing and has occurred in phases. When the study began, the types of HCP interacting with patients known to be positive for SARS-CoV-2 were limited per hospital policy, so the groups that were providing direct in-person care were targeted for recruitment first (physicians, nurses, nursing assistants, respiratory therapists, etc). As the pandemic continued, hospital policies shifted, and ancillary staff (interpreters, food services, environmental services, patient transporters, etc) were again entering patient rooms, so our recruitment efforts also shifted to include these groups. Specifically, we used the same recruitment strategies described above but targeted them to the support staff employee groups. In addition, we performed additional outreach to the environmental services group, attending shift huddles and rounding with management multiple times in person.

As of December 31, 2020, we have enrolled a total of 211 HCP participants. Of these, 65 (30.8%) are male and 146 (69.2%) are female. We have also enrolled 53 household participants from 37 households. Of those 53 household participants, 16 (30%) are under 18 years old. Many types of HCP have enrolled in the study (see Table 1), with the majority being physicians and registered nurses.

**Table 1:** Demographics of health care personnel participants enrolled in the study as of December 31, 2020, (N=211).

Demographic			n (%)
**Sex**			
	Male	65 (30.8)	
	Female	146 (69.2)	
**Role at the** **University of North Carolina Medical Center**			
	Case management	1 (0.5)	
	Child life specialist	3 (1.4)	
	Clinical dietitian	2 (0.9)	
	Certified registered nurse anesthetist	5 (2.4)	
	Certified surgical technician I or II	4 (1.9)	
	Extracorporeal membrane oxygenation specialist	1 (0.5)	
	Environmental services	2 (0.9)	
	Food services	1 (0.5)	
	Front desk coordinator	1 (0.5)	
	Health unit coordinator	1 (0.5)	
	Interpreter	3 (1.4)	
	Licensed practical nurse	2 (0.9)	
	Midwife	2 (0.9)	
	Nurse aide or certified nursing assistant	5 (2.4)	
	Nurse practitioner	4 (1.9)	
	Patient transporter	3 (1.4)	
	Physical or occupational therapist	10 (4.7)	
	Physician	84 (39.8)	
	Physician assistant	4 (1.9)	
	Radiology technologist	3 (1.4)	
	Respiratory Diagnostic Center swabber	1 (0.5)	
	Registered nurse	56 (26.5)	
	Respiratory therapist	10 (4.7)	
	Speech therapist	1 (0.5)	
	Ultrasound technologist	2 (0.9)	

A total of 86.3% (182/211) of participants have opted into the Bluetooth contact tracking, while 90.0% (190/211) of participants have opted into the NELF sample collection substudy. Thus far, 45 participants have withdrawn prior to 12 weeks of participation due to schedule constraints.

## Discussion

In this article, we describe the protocol for a multifaceted, longitudinal, observational cohort study to obtain crucial information about the risk of SARS-CoV-2 infection among HCP and their household contacts. There are several novel aspects to the study design that will maximize its impact. First, the cohort of HCP will be very well-characterized because of (1) frequent sampling, especially during the first 12 weeks, to assess for infection and/or seroconversion and (2) the depth and breadth of information collected through electronic questionnaires regarding clinical symptoms, potential exposures to SARS-CoV-2, occupational activities, PPE access and use, perceptions of the epidemic, and mental health. As we intentionally designed the study to include many different types of HCP to maximize the generalizability of our results, we have applied careful measurement where we can to identify occupational roles and activities so these can be accounted for in our analyses. Second, the use of Bluetooth beacon contact tracking is a robust methodology that will complement the self-reported exposure data. Finally, our study is unique in that it is enrolling both HCP and a randomly selected subset of linked households.

There are a few potential limitations to our study. First, this study design employs frequent in-person study visits and electronic contact throughout the study period. We designed the study this way intentionally, seeking to obtain highly granular and detailed information about risks and exposures among our study participants. There is a chance that this level of participation may limit our ability to reach our recruitment goal of 300 individuals. To offset this burden, after the first 12 weeks of the study, participants transition to having only monthly study visits and one survey every 2 weeks. Second, it is possible that the people who chose to enroll in this type of longitudinal study may be more attuned to the COVID-19 pandemic and, therefore, more focused on preventative measures. This could induce selection bias. We plan to compare characteristics of our study population to those that are available for our target population—all HCP at UNCMC—and consider weighting, if appropriate, based on that evaluation.

Through this study, we will generate important data about the incidence of SARS-CoV-2 infection among frontline HCP, their workplace contact network, transmission to their household members, and trends in incidence over time. We will also evaluate the accuracy and feasibility of using a minimally invasive, point-of-care rapid test to assess for seroconversion. Together, this information will be invaluable in determining the effectiveness of active surveillance programs to reduce nosocomial transmission and the need for additional nonpharmaceutical interventions to protect HCP and their families.

## Appendix

Multimedia Appendix 1 Baseline survey for health care personnel.

Multimedia Appendix 2 Daily survey for health care personnel.

Multimedia Appendix 3 Biweekly survey for health care personnel.

Multimedia Appendix 4 Biweekly survey for health care personnel (weeks 12, 24, and 36 only).

Multimedia Appendix 5 Instructions for participant self-collection of nasal epithelial lining fluid (NELF) samples.

Multimedia Appendix 6 Baseline survey for household participants.

Multimedia Appendix 7 Weekly survey for household participants.

Multimedia Appendix 8 Day 21 survey for household participants.

Multimedia Appendix 9 COVID-19 health care personnel (HCP) study laboratory protocol.

## Abbreviations

BLE: Bluetooth Low Energy
CDC: US Centers for Disease Control and Prevention
DBS: dried blood spots
ED: Emergency Department
ELISA: enzyme-linked immunosorbent assay
HCP: health care personnel
Ig: immunoglobulin
MICU: Medical Intensive Care Unit
MTNS: mid-turbinate nasal swab
NELF: nasal epithelial lining fluid
PBMC: peripheral blood mononuclear cell
PCR: polymerase chain reaction
PPE: personal protective equipment
PTSD: posttraumatic stress disorder
RDC: Respiratory Diagnostic Center
RDT: rapid diagnostic test
REDCap: Research Electronic Data Capture
RSSI: received signal strength indicator
UNC: University of North Carolina
UNCMC: University of North Carolina Medical Center
UUID: universally unique identifier
