# Supplementary material for: SARS-CoV-2 Infection in Health Care Personnel and Their Household Contacts at a Tertiary Academic Medical Center: Protocol for a Longitudinal Cohort Study
Source: JMIR Res Protoc. 2021 Apr 30;10(4):e25410. doi: 10.2196/25410 (PMC8092024; doi:10.2196/25410)
Supplement: Multimedia Appendix 1 [file resprot_v10i4e25410_app1.pdf]

Appendix 1: Baseline Survey for Healthcare Personnel

Please provide the following personal and health information.

1. What is your sex?
   - Female
   - Male

2. What is your race? *Select all that apply*
   - American Indian or Alaska Native
   - Asian
   - Black or African American
   - Native Hawaiian or Pacific Islander
   - White
   - Other

   If question 2 = Other:
   2.1. By what other race do you identify?

3. What is your ethnicity?
   - Hispanic or Latinx
   - Not Hispanic or Latinx
   - Other

4. What is your permanent address? Please provide the address of your household residence (not a PO box). We will use this information to send COVID-19 testing materials to your household if you are selected for further testing during this study.

5. Are you currently enrolled in any of the following scientific studies? *Select all that apply*
   - Not enrolled in any other studies
   - Enrolled in HERO trial of hydroxychloroquine
   - Enrolled in some other scientific study

   If question 5= Enrolled in some other scientific study:
   5.1. Please enter the name (or a brief description, if you don't know the name) of the other study you are enrolled in.

6. What is your role in UNC Health?
   - Physician
   - Physician assistant
   - Nurse practitioner
   - Registered nurse
   - Pharmacist
   - Physical/occupational therapist
   - Radiology technician
   - Environmental services
   - Food services
   - Laboratory staff

Please do not reuse or reproduce surveys without proper citation

Other

If question 6=Other:
6.1. What other role do you have in UNC Health?

If question 6 =Physician, physician assistant, or nurse practitioner:
6.1.1. What clinical department are you in?

Anesthesiology
Dermatology
Emergency Medicine
Family Medicine
Medicine
Neurology
Neurosurgery
Obstetrics and Gynecology
Ophthalmology
Orthopaedics
Otolaryngology/Head & Neck Surgery
Pathology and Laboratory Medicine
Pediatrics
Physical Medicine and Rehabilitation
Psychiatry
Radiation Oncology
Radiology
Surgery
Urology
Other

If question 6.2=Medicine:
6.1.1.1. What division of the Department of Medicine are you in?

Cardiology
Endocrinology & Metabolism
Gastroenterology & Hepatology
General Medicine and Clinical Epidemiology
Geriatric Medicine Hematology/Oncology
Hospital Medicine
Infectious Diseases
Nephrology & Hypertension
Pulmonary Diseases & Critical Care Medicine
Rheumatology, Allergy & Immunology

7. How many years have you worked in this role at UNC Health?

8. Do you currently smoke cigarettes, cigars, or a pipe on a daily basis? – Yes/No

If 8=Yes:

Please do not reuse or reproduce surveys without proper citation

Appendix 1: Baseline Survey for Healthcare Personnel

8.1. How old were you when you first started to smoke fairly regularly?

8.2. What is the average number of cigarettes smoked per day since you began smoking?

None
1-10
11-20
21-30
31-40
41-50
51-60
61 or more

8.3. What is the average number of cigars smoked per day since you began smoking?

None
1
2
3
4
5 or more

8.4. What is the average number of bowls of tobacco smoked per day since you began smoking?

None
1
2
3
4
5 or more

9. Did you previously smoke cigarettes, cigars, or a pipe on a daily basis? – Yes/No

If 9= Yes:

9.1. How old were you when you first started to smoke fairly regularly?

9.2. How many years has it been since you quit smoking?

9.3. What was the average number of cigarettes smoked per day when you were smoking?

None
1-10
11-20
21-30
31-40
41-50
51-60
61 or more

9.4. What was the average number of cigars smoked per day when you were smoking?

None

Please do not reuse or reproduce surveys without proper citation

1
2
3
4
5 or more

9.5. What was the average number of bowls of tobacco smoked per day when you were smoking?
None
1
2
3
4
5 or more

10. Do you currently use electronic cigarettes (e-cigarettes, vaping)? – Yes/No

If 10=Yes
10.1. How old were you when you first started to use electronic cigarettes fairly regularly?

10.2. What is the average number of e-cigarette (or other vaping product) puffs you inhale per day?
0-25
26-50
51-75
76-100
101-125
126-150
151-175
176-200
201-225
226-250
251 more

11. Did you previously use electronic cigarettes (e-cigarettes, vaping)? – Yes/No

If 11=Yes:
11.1. How old were you when you first started to use electronic cigarettes fairly regularly?

11.2. How many years has it been since you quit using electronic cigarettes?

11.3. What was the average number of e-cigarette (or other vaping product) puffs you inhaled per day?
0-25
26-50
51-75
76-100
101-125
126-150
151-175

Please do not reuse or reproduce surveys without proper citation

176-200
201-225
226-250
251 more

12. Do you currently drink alcohol at least once a week? – Yes/No

If 12=Yes:

12.1. How old were you when you first started to drink alcohol fairly regularly?

12.2. On how many weekdays (Monday through Friday) do you usually drink alcohol?

0
only occasionally
1
2
3
4
5

If 12.2 > 0:

12.2.1. When you drink on a weekday (Monday through Friday), how many drinks do you usually have? (One drink is equal to 5 ounces of wine, 12 ounces of beer, or 1.5 ounces of liquor)

1
2
3
4
5
6
7 or more

12.3. On how many weekend days (Saturday and Sunday) do you usually drink alcohol?

0
only occasionally
1
2

If 12.3 > 0:

12.3.1. When you drink on a weekend day (Saturday and Sunday), how many drinks do you usually have? (One drink is equal to 5 ounces of wine, 12 ounces of beer, or 1.5 ounces of liquor)

1
2
3
4
5
6
7 or more

Please do not reuse or reproduce surveys without proper citation

If 12 = No:
12.4. Did you previously drink alcohol at least once a week? – Yes/No

If 12.4=Yes:
12.4.1. How old were you when you first started to drink alcohol fairly regularly?

12.4.2. How many years has it been since you quit drinking alcohol?

12.4.3. On how many weekdays (Monday through Friday) did you usually drink alcohol?
0
only occasionally
1
2
3
4
5

If 12.4.3 > 0:
12.4.3.1. When you drank on a weekday (Monday through Friday), how many drinks did you usually have? (One drink is equal to 5 ounces of wine, 12 ounces of beer, or 1.5 ounces of liquor)
1
2
3
4
5
6
7 or more

12.4.4. On how many weekend days (Saturday and Sunday) did you usually drink alcohol?
0
only occasionally
1
2

If 12.4.4 > 0:
12.4.4.1. When you drank on a weekend day (Saturday and Sunday), how many drinks did you usually have? (One drink is equal to 5 ounces of wine, 12 ounces of beer, or 1.5 ounces of liquor)
1
2
3
4
5
6
7 or more

Please do not reuse or reproduce surveys without proper citation

13. At least once a week, do you engage in regular activity like brisk walking, jogging, bicycling, swimming, etc. long enough to work up a sweat, get your heart thumping, or get out of breath? – Yes/No

If 14=Yes:

13.1. On average, how many days per week do you engage in this kind of exercise?
- 1
- 2
- 3
- 4
- 5
- 6
- 7

13.2. On average, how many minutes per day do you engage in this kind of exercise?
- 0-20
- 21-40
- 41-60
- 61 or more

13.3. When you are exercising in your usual fashion, how would you rate your average level of exertion (degree of effort)?
- Easy / Warm-up
- Medium (can hold a conversation) / Aerobic Development
- Hard (but you can push yourself to continue) / Aerobic Endurance
- Very Hard (cannot hold a conversation) / Anaerobic Endurance
- Extremely Hard (out of breath, your body wants to stop) / Speed, Power

14. Have you ever received a diagnosis of any of the following? - Select Yes or No for each diagnosis

14.1. Asthma
14.2. Diabetes
14.3. Hypertension
14.4. Cardiovascular disease
14.5. Cancer
14.6. Chronic lung or respiratory disease
14.7. Other chronic condition

If 14.7=Yes:

14.7.1. Please identify what other chronic medical condition you have been diagnosed with.

15. Have you previously been diagnosed with COVID-19? – Yes/No

If 15=Yes:

15.1. When were you previously diagnosed with COVID-19? Please provide your best guess as to your date of testing, or if not tested for COVID-19, then your best guess as to the date when you were diagnosed by a clinician.

Please do not reuse or reproduce surveys without proper citation

15.2. Please provide your best guess of how you were exposed to, and infected with, COVID-19.
- Travel (airport, bus station, etc.)
- Community transmission (retail setting, etc.)
- Household contact (sick household or family member, etc.)
- Occupational (clinic or hospital exposure, etc.)
- Other
- Unknown

If 15.2 =Other:
15.2.1. Please explain what other route of exposure you experienced.

If 15= No:
15.3. Whether or not you were tested and diagnosed, have you previously experienced symptoms that made you believe you were infected with COVID-19? – Yes/No

If 15.3=Yes:
15.3.1. When did you first experience symptoms that made you believe you were infected with COVID-19? Please provide your best guess of the date when you first believed that you may have had COVID-19.

15.3.2. Please provide your best guess of how you may have been exposed to, and infected with, COVID-19.
- Travel (airport, bus station, etc.)
- Community transmission (retail setting, etc.)
- Household contact (sick household or family member, etc.)
- Occupational (clinic or hospital exposure, etc.)
- Other
- Unknown

If 15.3.2 = Other:
15.3.2.1. Please explain what other route of exposure you experienced.

16. During the last two weeks, have you experienced any of the following symptoms? – Select Yes or No for each symptom

16.1. fever (measured by thermometer or self-diagnosed)
16.2. cough (new or worsening)
16.3. shortness of breath (new or worsening)
16.4. fatigue (new tiredness doing normal activities)
16.5. body aches
16.6. headache
16.7. diarrhea
16.8. sore throat
16.9. itchy, pink, or painful eyes
16.10. runny nose or congestion
16.11. changes in your sense of smell or taste
16.12. new rash
16.13. repeated shaking with chills

Please do not reuse or reproduce surveys without proper citation

If any question 16.1 to 16.13=Yes, questions 17 to 20 display. If none of 16.1 to 16.13=Yes, survey skips to question 21.

17. When did the symptoms reported above first start?

18. What did you do in response to the symptoms reported above?
    - Nothing
    - Took over the counter medication
    - Called Occupational Health
    - Visited Occupational Health
    - Called Respiratory Diagnostic Center
    - Visited Respiratory Diagnostic Center
    - Called outside clinic
    - Visited outside clinic
    - Other

If 18=Other:

18.1. Please specify what other action you took in response to your symptoms.

19. Given the symptoms you reported, how worried were you that you may have been infected with COVID-19?
    - Not at all worried
    - Slightly worried
    - Very worried
    - Extremely worried

20. Given the symptoms you reported, did you attempt to receive a COVID-19 test? – Yes/No

If 20=Yes:

20.1. How many days passed between your first reported symptoms and your first attempt to receive a COVID-19 test?
- 0 days
- 1 day
- 2 days
- 3 days
- 4 days
- 5 days
- 6 days
- 7 days
- more than 7 days

20.2. How difficult was it to actually receive a COVID-19 test?
- Not at all difficult
- Slightly difficult
- Very difficult
- Extremely difficult

Please do not reuse or reproduce surveys without proper citation

21. Did you receive a test for COVID-19 during the last two weeks in response to the symptoms you reported above or for any other reason not reported? – Yes/No

    If 21=Yes:
    21.1. Where were you tested for COVID-19?

    21.2. What was the result of your COVID-19 test?
    - Result still pending
    - Positive for COVID-19
    - Negative for COVID-19
    - Inconclusive result

22. Which of the following practices has your household employed to limit the risk of being exposed to COVID-19 during the COVID-19 pandemic in North Carolina? Select Always, Most of the time, Half of the time, Less than half of the time or Never for each practice.
    22.1. Wearing face masks inside of the home
    22.2. Wearing face masks outside of the home
    22.3. Washing hands and/or using sanitizer frequently
    22.4. Staying at least 6 feet away from others
    22.5. Avoiding large gatherings, public spaces, or crowds
    22.6. Avoiding contact with people who could be high risk
    22.7. Avoiding food from restaurants, including takeout
    22.8. Working or studying at home instead of going into an office/classroom
    22.9. Avoiding shaking hands or touching people
    22.10. Staying home when sick
    22.11. Wiping down surfaces with disinfectant
    22.12. Cancelling or postponing planned travel for work
    22.13. Cancelling or postponing travel for pleasure
    22.14. Cancelling or postponing personal or social activities
    22.15. Cancelling doctor
    22.16. Stockpiling food or water
    22.17. Following government guidelines or rules to shelter in place (i.e. staying at home, limiting non-essential travel, etc.)

Please provide the following information about your household contacts.

23. How many additional people (not including yourself) live or spend a significant amount of time (more than 40 hours per week on average) in your primary residence?
    - 0
    - 1
    - 2
    - 3
    - 4
    - 5
    - 6
    - 7 or more

Please do not reuse or reproduce surveys without proper citation

Appendix 1: Baseline Survey for Healthcare Personnel

24. Do you have any pets in your primary residence? – Yes/No

If 24=Yes:
24.1. What pet(s) do you have in your primary residence?
- Dog(s)
- Cat(s)
- Other(s)

Questions 27 to 33 repeated for each person identified in question 25, up to 7 people
25. What is your relationship to this person?
- Partner or spouse
- Child
- Parent
- Sibling
- Other family member
- In-home childcare provider or other caregiver
- Other

If 25=Other:
25.1. Please specify your relationship with this person

26. What is this person's age?

27. Does this person also work in a healthcare facility? Yes/No

If 27=Yes:
27.1. What is this person's position at their job in a healthcare facility?
- Physician
- Physician assistant
- Nurse practitioner
- Registered nurse
- Pharmacist
- Physical/occupational therapist
- Radiology technician
- Environmental services
- Food services
- Laboratory staff
- Other

28. Does this person work in any of the other high-risk facilities?
- Nursing home
- Prison
- Assisted living
- Other congregate residential facility
- Does not work in a high-risk facility

Please do not reuse or reproduce surveys without proper citation

Appendix 1: Baseline Survey for Healthcare Personnel

29. Has this person come in contact with anyone suspected to be positive for COVID-19? - Yes/No

30. Has this person come in contact with anyone known to be positive for COVID-19? - Yes/No

31. Has this person had any symptoms (cough, fever, difficulty breathing, fatigue, body aches, etc.) consistent with COVID-19 in the last two weeks? - Yes/No

If 31=Yes:

31.1. When did their symptoms consistent with COVID-19 first start?

31.2. Has this person been tested for COVID-19? - Yes/No

If 31.2 =Yes,

31.2.1. Where was this person tested for COVID-19?

31.2.2. What was the result of this person's COVID-19 test?

- Result still pending
- Positive for COVID-19
- Negative for COVID-19
- Inconclusive result

32. Are you currently staying at your primary residence? - Yes/No

If 32 =No:

32.1. In what temporary residence are you currently staying?

- Relative
- Friend
- AirBnB or other non-hotel rental
- Hotel
- Other

32.2. Why are you staying in a temporary residence?

- Convenience to UNC work location
- Travel restrictions
- Fear of spreading COVID-19 to primary household
- Other

32.3. How many additional people (not including yourself) live or spend a significant amount of time (more than 40 hours per week on average) in your temporary residence?

- 0
- 1
- 2
- 3
- 4
- 5
- 6
- 7 or more

Please do not reuse or reproduce surveys without proper citation

32.4. Do you have any pets in your temporary residence? - Yes/No

If 32.4 =Yes:
32.4.1. What pet(s) do you have in your temporary residence?
Dog(s)
Cat(s)
Other(s)

Questions 33 to 39 repeated for each person identified in question 32.3, up to 7 people
33. What is your relationship to this person?
Partner or spouse
Child
Parent
Sibling
Other family member
In-home childcare provider or other caregiver
Other

If 33=Other:
33.1. Please specify your relationship with this person.

34. What is this person's age?

35. Does this person also work in a healthcare facility? - Yes/No

If 35=Yes:
35.1. What is this person's position at their job in a healthcare facility?
Physician
Physician assistant
Nurse practitioner
Registered nurse
Pharmacist
Physical/occupational therapist
Radiology technician
Environmental services
Food services
Laboratory staff
Other

36. Does this person work in any of the other high-risk facilities?
Nursing home
Prison
Assisted living
Other congregate residential facility
Foes not work in a high-risk facility

Please do not reuse or reproduce surveys without proper citation

Appendix 1: Baseline Survey for Healthcare Personnel

37. Has this person come in contact with anyone suspected to be positive for COVID-19? - Yes/No

38. Has this person come in contact with anyone known to be positive for COVID-19? - Yes/No

39. Has this person had any symptoms (cough, fever, difficulty breathing, fatigue, body aches, etc.) consistent with COVID-19 in the last two weeks?- Yes/No

If 39= Yes:

39.1. When did their symptoms consistent with COVID-19 first start?

39.2. Has this person been tested for COVID-19?

If 39.2=Yes:

39.2.1. Where was this person tested for COVID-19?

39.2.2. What was the result of this person's COVID-19 test?

Result still pending
Positive for COVID-19
Negative for COVID-19
Inconclusive result

Please provide the following information about your potential exposures to COVID-19.

40. How many hours have you worked in any UNC hospital or other clinical setting over the last 2 weeks?

41. How many hours have you cared for patients with COVID-19 at UNC Hospital over the last 2 weeks?

If 41 >0:

41.1. What COVID-19 teams did you work on during the last 2 weeks?

Med Z admitting
Med Z rounding
Med Z cross-cover
COVID ICU
COVID ID
Other
None of the above

If 41.1 =Other:

41.1.1. Please specify what other COVID-19 team you worked on during the last 2 weeks.

41.2. What COVID-19 units did you work on during the last 2 weeks?

ED D Bay
RDC
MICU
6BT
MPCU

Please do not reuse or reproduce surveys without proper citation

8BT
Other

If 41.2 = Other:
41.2.1. Please specify what other COVID-19 unit you worked on during the last 2 weeks.

The following questions refer to contacts with patients suspected or known to be positive for COVID-19.

42. How often have you come in contact with a patient who is suspected or known to be positive for COVID-19 during the last two weeks? (Example: examining one patient 5 times and another patient 2 times counts as 7 total contacts)
    Never
    1-5 times
    6-10 times
    11-15 times
    16-20 times
    21-25 times
    26-30 times
    31-35 times
    36 or more times

If 42 is NOT never, questions 43 – 50 are displayed. If 42=Never, the survey skips to question 51.

43. Please estimate the total number of hours spent in a room with a patient suspected or known to be positive for COVID-19 during the last two weeks. (Example: examining one patient for 15 minutes and two patients for 30 minutes each is a total of 1 hour and 15 minutes of contact, or 1-1.9 hours)
    <1
    1-1.9 hours
    2-2.9 hours
    3-3.9 hours
    4-4.9 hours
    5-9.9 hours
    10-14.9 hours
    15-19.9 hours
    20-29.9 hours
    30-39.9 hours
    40 or more hours

44. Please estimate the number of times you performed each of the following non-aerosol generating procedures with a patient suspected or known to be positive for COVID-19 during the last two weeks.
    Select one for each procedure: 0 times, 1 time, 2 times, 3 times, 4 times, 5 times , 6-10 times, 11-15 times, 16-20 times, 21-30 times, 31 or more times
    44.1. measuring vital signs
    44.2. collecting a medical history
    44.3. performing a physical exam
    44.4. providing medication
    44.5. bathing or cleaning
    44.6. lifting or positioning

Please do not reuse or reproduce surveys without proper citation

44.7. emptying bedpan
44.8. changing linens
44.9. cleaning the room
44.10. inserting a peripheral line
44.11. inserting a central line
44.12. drawing arterial blood gas
44.13. drawing blood 0, 0 times
44.14. manipulating an oxygen mask or tubing
44.15. manipulating a ventilator or tubing
44.16. delivering high-flow oxygen
44.17. collecting respiratory specimens

45. Please estimate the number of times you performed each of the following aerosol generating procedures with a patient suspected or known to be positive for COVID-19 during the last two weeks.
Select one for each procedure: 0 times, 1 time, 2 times, 3 times, 4 times, 5 times , 6-10 times, 11-15 times, 16-20 times, 21-30 times, 31 or more times
45.1. performing airway suctioning
45.2. noninvasive ventilation (BiPaP, CPAP)
45.3. performing manual (bag) ventilation
45.4. providing nebulizer treatment
45.5. breaking the ventilation circuit
45.6. assisting in sputum induction
45.7. performing or assisting in intubation
45.8. present or in the room during intubation
45.9. performing or assisting in bronchoscopy
45.10. present or in the room during bronchoscopy

46. How often have you had access to the following PPE during interactions with patients suspected or known to be positive for COVID-19 during the last two weeks?
Select one for each item: all of the time (100%), most of the time (75%), half of the time (50%), one-quarter of the time (25%), never (0%)
46.1. gown
46.2. gloves
46.3. eye shield
46.4. face mask

47. What type of face mask did you use most frequently for encounters with patients suspected or known to be positive for COVID-19 during the last two weeks?
Surgical ear loop
Surgical tie
N95
Homemade mask
Other

If 47= Other,
47.1. What other type of mask did you use most frequently?

Please do not reuse or reproduce surveys without proper citation

48. Did you ever reuse face masks used with patients suspected or known to be positive for COVID-19 during the last two weeks?
    Yes
    No

49. During your interactions with patients suspected or known to be positive for COVID-19 during the last two weeks, how often was the PATIENT wearing a mask?
    all of the time (100%)
    most of the time (75%)
    half of the time (50%)
    one-quarter of the time (25%)
    never (0%)

50. During your interactions with patients suspected or known to be positive for COVID-19 during the last two weeks, how often were you NOT wearing eye protection when the patient was also NOT wearing a mask?
    all of the time (100%)
    most of the time (75%)
    half of the time (50%)
    one-quarter of the time (25%)
    never (0%)

The following questions refer to contacts with patients NOT known or suspected to be positive for COVID-19.

51. How often have you come in contact with a patient who is NOT known or suspected to be positive for COVID-19 during the last two weeks?
    Never
    1-5 times
    6-10 times
    11-15 times
    16-20 times
    21-25 times
    26-30 times
    31-35 times
    36 or more times

If 51 is NOT Never, questions 52 to 59 display. If 51 = Never, the survey skips to question 60.

52. Please estimate the total number of hours spent in a room with a patient NOT known or suspected to be positive for COVID-19 during the last two weeks.
    <1
    1-1.9 hours
    2-2.9 hours
    3-3.9 hours
    4-4.9 hours
    5-9.9 hours
    10-14.9 hours

Please do not reuse or reproduce surveys without proper citation

15-19.9 hours
20-29.9 hours
30-39.9 hours
40 or more hours

53. Please estimate the number of times you performed each of the following non-aerosol generating procedures with a patient NOT known or suspected to be positive for COVID-19 during the last two weeks. Select one for each procedure: 0 times, 1 time, 2 times, 3 times, 4 times, 5 times , 6-10 times, 11-15 times, 16-20 times, 21-30 times, 31 or more times
    53.1. measuring vital signs
    53.2. collecting a medical history
    53.3. performing a physical exam
    53.4. providing medication
    53.5. bathing or cleaning
    53.6. lifting or positioning
    53.7. emptying bedpan
    53.8. changing linens
    53.9. cleaning the room
    53.10. inserting a peripheral line
    53.11. inserting a central line
    53.12. drawing arterial blood gas
    53.13. drawing blood
    53.14. manipulating an oxygen mask or tubing
    53.15. manipulating a ventilator or tubing
    53.16. delivering high-flow oxygen
    53.17. collecting respiratory specimens

54. Please estimate the number of times you performed each of the following aerosol generating procedures with a patient NOT known or suspected to be positive for COVID-19 during the last two weeks. Select one for each procedure: 0 times, 1 time, 2 times, 3 times, 4 times, 5 times , 6-10 times, 11-15 times, 16-20 times, 21-30 times, 31 or more times
    54.1. performing airway suctioning
    54.2. noninvasive ventilation (BiPaP, CPAP)
    54.3. performing manual (bag) ventilation
    54.4. providing nebulizer treatment
    54.5. breaking the ventilation circuit
    54.6. assisting in sputum induction
    54.7. performing or assisting in intubation
    54.8. present or in the room during intubation
    54.9. performing or assisting in bronchoscopy
    54.10. present or in the room during bronchoscopy

55. How often have you had access to the following PPE during interactions with patients NOT known or suspected to be positive for COVID-19 during the last two weeks? Select one for each item: all of the time (100%), most of the time (75%), half of the time (50%), one-quarter of the time (25%), never (0%)
    55.1. gown

Please do not reuse or reproduce surveys without proper citation

55.2. gloves
55.3. eye shield
55.4. face mask

56. What type of face mask did you use most frequently for encounters with patients NOT known or suspected to be positive for COVID-19 during the last two weeks?
    - Surgical ear loop
    - Surgical tie
    - N95
    - Homemade mask
    - Other

If 56= Other,
56.1. What other type of mask did you use most frequently?

57. Did you ever reuse face masks used with patients NOT known or suspected to be positive for COVID-19 during the last two weeks?
    - Yes
    - No

58. During your interactions with patients NOT known or suspected to be positive for COVID-19 during the last two weeks, how often was the PATIENT wearing a mask?
    - all of the time (100%)
    - most of the time (75%)
    - half of the time (50%)
    - one-quarter of the time (25%)
    - never (0%)

59. During your interactions with patients NOT known or suspected to be positive for COVID-19 during the last two weeks, how often were you NOT wearing eye protection when the patient was also NOT wearing a mask?
    - all of the time (100%)
    - most of the time (75%)
    - half of the time (50%)
    - one-quarter of the time (25%)
    - never (0%)

Please provide the general following information about PPE and sanitation.

60. How many times were you notified that you donned or doffed PPE incorrectly during the last two weeks?
    - 0
    - 1
    - 2
    - 3
    - 4
    - 5
    - 6
    - 7

Please do not reuse or reproduce surveys without proper citation

8
9
10 or more

If 60 >0:
60.1. Please explain what happened when you donned/doffed PPE incorrectly.

61. How many times did you miss a hand or glove hygiene step during the PPE donning and doffing procedure during the last two weeks?
    0 times
    1 time
    2 times
    3 times
    4 times
    5 times
    6-10 times
    11-15 times
    16-20 times
    21 or more times

62. If you worked on a floor where the COVID patients are cohorted and PPE donning and doffing occurs at the entrance to the ward, how many times did you forget to perform hand or glove hygiene between patient rooms?
    0 times
    1 time
    2 times
    3 times
    4 times
    5 times
    6-10 times
    11-15 times
    16-20 times
    21 or more times
    NA (did not work on a ward with cohorted COVID patients)

63. How often have you worn a face mask outside of work during the last two weeks?
    all of the time (100%)
    most of the time (75%)
    half of the time (50%)
    one-quarter of the time (25%)
    never (0%)

64. How often have you worn a face mask at home during the last two weeks?
    all of the time (100%)
    most of the time (75%)
    half of the time (50%)
    one-quarter of the time (25%)

Please do not reuse or reproduce surveys without proper citation

never (0%)

65. During the last two weeks, how often have you been bothered by the following problems?
Select one for each problem: not at all, several days, more than half of the days, nearly every day
65.1. Feeling nervous, anxious, or on edge
65.2. Not being able to stop or control worrying
65.3. Worrying too much about different things
65.4. Trouble relaxing
65.5. Being so restless that it's hard to sit still
65.6. Becoming easily annoyed or irritable
65.7. Feeling afraid as if something awful might happen

If any from 65.1 to 65.7 is NOT "not at all":
65.7.1. How difficult have these problems made it for you to do your work, take care of things at home, or get along with other people?
not difficult
somewhat difficult
very difficult
extremely difficult

66. During the last two weeks, how often have you been bothered by the following problems?
Select one for each item: not at all, several days, more than half of the days, nearly every day
66.1. Little interest or pleasure in doing things
66.2. Feeling down, depressed, or hopeless
66.3. Trouble falling or staying asleep, or sleeping too much
66.4. Feeling tired or having little energy
66.5. Poor appetite or overeating
66.6. Feeling bad about yourself, or that you are a failure or have let yourself or your family down
66.7. Trouble concentrating on things, such as reading the newpaper or watching television
66.8. Moving or speaking so slowly that other people could have noticed, or the opposite - being so fidgety or restless that you have been moving around a lot more than usual
66.9. Thoughts that you would be better off dead, or of hurting yourself

If any question from 66.1 to 66.9 is NOT "not at all":
66.9.1. How difficult have these problems made it for you to do your work, take care of things at home, or get along with other people?
0, not difficult
1, somewhat difficult
2, very difficult
3, extremely difficult

67. In your life, have you ever had any experience that was so frightening, horrible, or upsetting that, in the past month, you: Select Yes or No for each item
67.1. Have had nightmares about it or thought about it when you did not want to
67.2. Tried hard not to think about it or went out of your way to avoid situations that reminded you of it

Please do not reuse or reproduce surveys without proper citation

67.3. Were constantly on guard, watchful, or easily startled
67.4. Felt numb or detached from others, activities, or your surroundings

68. During the last two weeks, have you experienced the following due to the COVID-19 pandemic? Select Yes or No for each time
    68.1. I believed that my job was putting me at great risk
    68.2. I felt extra stress at work
    68.3. I was afraid of falling ill with COVID-19
    68.4. I felt I had little control over whether I would get infected or not
    68.5. I thought I would be unlikely to survive if I were to get COVID-19
    68.6. I thought about resigning because of COVID-19
    68.7. I was afraid I would pass COVID-19 on to others
    68.8. My family and friends were worried that they might get infected through me
    68.9. People avoided my family because of my work
    68.10. I was willing to accept the risks involved because I wanted to help the COVID-19 patients

69. The questions in this scale ask you about your feelings and thoughts during the last month. Select one for each question: never, almost never, sometimes, fairly often, very often
    69.1. In the last month, how often have you been upset because of something that happened unexpectedly?
    69.2. In the last month, how often have you felt that you were unable to control the important things in your life?
    69.3. In the last month, how often have you felt nervous and stressed?
    69.4. In the last month, how often have you felt confident about your ability to handle your personal problems?
    69.5. In the last month, how often have you felt that things were going your way?
    69.6. In the last month, how often have you found that you could not cope with all of the things that you had to do?
    69.7. In the last month, how often have you been able to control irritations in your life?
    69.8. In the last month, how often have you felt that you were on top of things?
    69.9. In the last month, how often have you been angered because of things that were outside of your control?
    69.10. In the last month, how often have you felt that difficulties were piling up so high that you could not overcome them?

Please do not reuse or reproduce surveys without proper citation
