# Supplementary material for: SARS-CoV-2 Infection in Health Care Personnel and Their Household Contacts at a Tertiary Academic Medical Center: Protocol for a Longitudinal Cohort Study
Source: JMIR Res Protoc. 2021 Apr 30;10(4):e25410. doi: 10.2196/25410 (PMC8092024; doi:10.2196/25410)
Supplement: Multimedia Appendix 2 [file resprot_v10i4e25410_app2.pdf]

Appendix 2: Daily Survey for Healthcare Personnel

1. Are you experiencing any of the following symptoms? – Select Yes or No for each symptom
   1.1. fever (measured by thermometer or self-diagnosed)
   1.2. cough (new or worsening)
   1.3. shortness of breath (new or worsening)
   1.4. fatigue (new tiredness doing normal activities)
   1.5. body aches
   1.6. headache
   1.7. diarrhea
   1.8. sore throat
   1.9. itchy, pink, or painful eyes
   1.10. runny nose or congestion
   1.11. changes in your sense of smell or taste
   1.12. new rash
   1.13. repeated shaking with chills

2. How many individuals did you come in close contact with today (defined as less than 6 ft)?
   Less than 5
   5-10
   11-16
   17+

3. How many hours have you worked in any UNC hospital or other clinical setting over the last 24 hours?

   If 3 is >0:
   3.1. How many hours have you cared for patients with COVID-19 at UNC Hospital over the last 24 hours?

   If 3.1 is >0:
   3.1.1. What COVID-19 teams did you work on during the last 24 hours? Select all that apply
   Med Z admitting
   Med Z rounding
   Med Z cross-cover
   COVID ICU
   COVID ID
   Other
   None of the above

   If 3.1.1 = Other:
   3.1.1.1. Please specify what other COVID-19 team you worked on during the last 24 hours.

   3.1.2. What COVID-19 units did you work on during the last 24 hours? Select all that apply
   ED D Bay
   RDC
   MICU
   6BT
   MPCU

Please do not reuse or reproduce surveys without proper citation

8BT
Other

If 3.1.2 = Other:
3.1.2.1. Please specify what other COVID-19 unit you worked on during the last 24 hours.

The following questions refer to contacts with patients suspected or known to be positive for COVID-19.

3.2. How often have you come in contact with a patient who is suspected or known to be positive for COVID-19 during the last 24 hours? (Example: examining one patient 5 times and another patient 2 times counts as 7 total contacts)

Never
1-5 times
6-10 times
11-15 times
16-20 times
21-25 times
26-30 times
31-35 times
36 or more times

If the answer to question 3.2 is NOT "Never," then questions 3.2.1-3.2.8 display. If the answer to question 3.2 is "Never," survey skips ahead to question 3.2.9.

3.2.1. Please estimate the total number of hours spent in a room with a patient suspected or known to be positive for COVID-19 during the last 24 hours. (Example: examining one patient for 15 minutes and two patients for 30 minutes each is a total of 1 hour and 15 minutes of contact, or 1-1.9 hours)

<1
1-1.9 hours
2-2.9 hours
3-3.9 hours
4-4.9 hours
5-9.9 hours
10-14.9 hours
15-19.9 hours
30-39.9hours
30-39.9 hours
40 or more hours

3.2.2. Please estimate the number of times you performed each of the following non-aerosol generating procedures with a patient suspected or known to be positive for COVID-19 during the last 24 hours. Select one for each procedure: 0 times, 1 time, 2 times, 3 times, 4 times, 5 times, 6-10 times, 11-15 times, 16-20 times, 21or more times

3.2.2.1. measuring vital signs
3.2.2.2. collecting a medical history
3.2.2.3. performing a physical exam

Please do not reuse or reproduce surveys without proper citation

Appendix 2: Daily Survey for Healthcare Personnel

- 3.2.2.4. providing medication
- 3.2.2.5. bathing or cleaning
- 3.2.2.6. lifting or positioning
- 3.2.2.7. emptying bedpan
- 3.2.2.8. changing linens
- 3.2.2.9. cleaning the room
- 3.2.2.10. inserting a peripheral line
- 3.2.2.11. inserting a central line
- 3.2.2.12. drawing arterial blood gas
- 3.2.2.13. drawing blood
- 3.2.2.14. manipulating an oxygen mask or tubing
- 3.2.2.15. manipulating a ventilator or tubing
- 3.2.2.16. delivering high-flow oxygen
- 3.2.2.17. collecting respiratory specimens
- 3.2.2.18. providing in-person interpretation

3.2.3. Please estimate the number of times you performed each of the following aerosol generating procedures with a patient suspected or known to be positive for COVID-19 during the last 24 hours. Select one for each procedure: 0 times, 1 time, 2 times, 3 times, 4 times, 5 times , 6-10 times, 11-15 times, 16-20 times, 21or more times

- 3.2.3.1. performing airway suctioning
- 3.2.3.2. noninvasive ventilation (BiPaP, CPAP)
- 3.2.3.3. performing manual (bag) ventilation
- 3.2.3.4. providing nebulizer treatment
- 3.2.3.5. breaking the ventilation circuit
- 3.2.3.6. assisting in sputum induction
- 3.2.3.7. performing or assisting in intubation
- 3.2.3.8. present or in the room during intubation
- 3.2.3.9. performing or assisting in bronchoscopy
- 3.2.3.10. present or in the room during bronchoscopy

3.2.4. How often have you had access to the following PPE during interactions with patients known or suspected to be positive for COVID-19 during the last 24 hours? Select one for each item: all of the time (100%), most of the time (75%), half of the time (50%), one-quarter of the time (25%), never (0%)

- 3.2.4.1. gown
- 3.2.4.2. gloves
- 3.2.4.3. eye shield
- 3.2.4.4. face mask

3.2.5. What type of face mask did you use most frequently for encounters with patients known or suspected to be positive for COVID-19 during the last two weeks?

Surgical ear loop
Surgical tie
N95
Homemade mask

Please do not reuse or reproduce surveys without proper citation

Other

3.2.6. Did you ever reuse face masks used with patients known or suspected to be positive for COVID-19 during the last 24 hours?

Yes
No

3.2.7. During your interactions with patients suspected or known to be positive for COVID-19 during the last 24 hours, how often was the PATIENT wearing a mask?

all of the time (100%)
most of the time (75%)
half of the time (50%)
one-quarter of the time (25%)
never (0%)

3.2.8. During your interactions with patients suspected or known to be positive for COVID-19 during the last 24 hours, how often were you NOT wearing eye protection when the patient was also NOT wearing a mask?

all of the time (100%)
most of the time (75%)
half of the time (50%)
one-quarter of the time (25%)
never (0%)

The following questions refer to contacts with patients NOT known or suspected to be positive for COVID-19.

3.2.9. How often have you come in contact with a patient who is NOT known or suspected to be positive for COVID-19 during the last 24 hours? (Example: examining one patient 5 times and another patient 2 times counts as 7 total contacts)

Never
1-5 times
6-10 times
11-15 times
16-20 times
21-25 times
26-30 times
31-35 times
36 or more times

If the answer to question 3.2.9 is NOT "Never," then questions 3.2.9.1-3.2.9.8 display

3.2.9.1. Please estimate the total number of hours spent in a room with a patient NOT known or suspected to be positive for COVID-19 during the last 24 hours. *Example: examining one patient for 15 minutes and two patients for 30 minutes each is a total of 1 hour and 15 minutes of contact, or 1-1.9 hours*

<1
1-1.9 hours

Please do not reuse or reproduce surveys without proper citation

2-2.9 hours
3-3.9 hours
4-4.9 hours
5-9.9 hours
10-14.9 hours
15-19.9 hours
20-29.9 hours
30-39.9 hours
40 or more hours

3.2.9.2. Please estimate the number of times you performed each of the following non-aerosol generating procedures with a patient NOT known or suspected to be positive for COVID-19 during the last 24 hours. Select one for each procedure: 0 times, 1 time, 2 times, 3 times, 4 times, 5 times , 6-10 times, 11-15 times, 16-20 times, 21 or more times

3.2.9.2.1. measuring vital signs
3.2.9.2.2. collecting a medical history
3.2.9.2.3. performing a physical exam
3.2.9.2.4. providing medication
3.2.9.2.5. bathing or cleaning
3.2.9.2.6. lifting or positioning
3.2.9.2.7. emptying bedpan
3.2.9.2.8. changing linens
3.2.9.2.9. cleaning the room
3.2.9.2.10. inserting a peripheral line
3.2.9.2.11. inserting a central line
3.2.9.2.12. drawing arterial blood gas
3.2.9.2.13. drawing blood
3.2.9.2.14. manipulating an oxygen mask or tubing
3.2.9.2.15. manipulating a ventilator or tubing
3.2.9.2.16. delivering high-flow oxygen
3.2.9.2.17. collecting respiratory specimens
3.2.9.2.18. providing in-person interpretation

3.2.9.3. Please estimate the number of times you performed each of the following aerosol generating procedures with a patient NOT known or suspected to be positive for COVID-19 during the last 24 hours. Select one for each procedure: 0 times, 1 time, 2 times, 3 times, 4 times, 5 times , 6-10 times, 11-15 times, 16-20 times, 21 or more times

3.2.9.3.1. performing airway suctioning
3.2.9.3.2. noninvasive ventilation (BiPaP, CPAP)
3.2.9.3.3. performing manual (bag) ventilation
3.2.9.3.4. providing nebulizer treatment
3.2.9.3.5. breaking the ventilation circuit
3.2.9.3.6. assisting in sputum induction
3.2.9.3.7. performing or assisting in intubation
3.2.9.3.8. present or in the room during intubation
3.2.9.3.9. performing or assisting in bronchoscopy

Please do not reuse or reproduce surveys without proper citation

3.2.9.3.10. present or in the room during bronchoscopy

3.2.9.4. How often have you had access to the following PPE during interactions with patients NOT known or suspected to be positive for COVID-19 during the last 24 hours? Select one for each item: all of the time (100%), most of the time (75%), half of the time (50%), one-quarter of the time (25%), never (0%)

3.2.9.4.1. gown
3.2.9.4.2. gloves
3.2.9.4.3. eye shield
3.2.9.4.4. face mask

3.2.9.5. What type of face mask did you use most frequently for encounters with patients NOT known or suspected to be positive for COVID-19 during the last 24 hours?

Surgical ear loop
Surgical tie
N95
Homemade mask
Other

3.2.9.6. Did you ever reuse face masks used with patients NOT known or suspected to be positive for COVID-19 during the last 24 hours?

Yes
No

3.2.9.7. During your interactions with patients NOT known or suspected to be positive for COVID-19 during the last 24 hours, how often was the PATIENT wearing a mask?

all of the time (100%)
most of the time (75%)
half of the time (50%)
one-quarter of the time (25%)
never (0%)

3.2.9.8. During your interactions with patients NOT known or suspected to be positive for COVID-19 during the last 24 hours, how often were you NOT wearing eye protection when the patient was also NOT wearing a mask?

all of the time (100%)
most of the time (75%)
half of the time (50%)
one-quarter of the time (25%)
never (0%)

Please do not reuse or reproduce surveys without proper citation
