# Supplementary material for: SARS-CoV-2 Infection in Health Care Personnel and Their Household Contacts at a Tertiary Academic Medical Center: Protocol for a Longitudinal Cohort Study
Source: JMIR Res Protoc. 2021 Apr 30;10(4):e25410. doi: 10.2196/25410 (PMC8092024; doi:10.2196/25410)
Supplement: Multimedia Appendix 3 [file resprot_v10i4e25410_app3.pdf]

Appendix 3: Biweekly Survey for Healthcare Personnel

1. Are you currently enrolled in any of the following scientific studies?
   - Not enrolled in any other studies
   - Enrolled in HERO trial of hydroxychloroquine
   - Enrolled in some other scientific study

   If 1 = Enrolled in some other scientific study:

   1.1. Please enter the name (or a brief description, if you don't know the name) of the other study you are enrolled in.

2. During the last two weeks, have you experienced any of the following symptoms? Select Yes or No for each symptom
   - 2.1. fever (measured by thermometer or self-diagnosed)
   - 2.2. cough (new or worsening)
   - 2.3. shortness of breath (new or worsening)
   - 2.4. fatigue (new tiredness doing normal activities)
   - 2.5. body aches
   - 2.6. headache
   - 2.7. diarrhea
   - 2.8. sore throat
   - 2.9. itchy, pink, or painful eyes
   - 2.10. runny nose or congestion
   - 2.11. changes in your sense of smell or taste
   - 2.12. new rash
   - 2.13. repeated shaking with chills

If any of 2.1-2.13 are answered Yes, questions 3-6 display. If all of 2.1-2.13 are No, survey skips to question 7.

3. When did the symptoms reported above first start?

4. What did you do in response to the symptoms reported above?
   - Nothing
   - Took over the counter medication
   - Called Occupational Health
   - Visited Occupational Health
   - Called Respiratory Diagnostic Center
   - Visited Respiratory Diagnostic Center
   - Called outside clinic
   - Visited outside clinic
   - Other

   If 4 = Other:

   4.1. Please specify what other action you took in response to your symptoms.

5. Given the symptoms you reported, how worried were you that you may have been infected with COVID-19?
   - Not at all worried
   - Slightly worried
   - Very worried

Please do not reuse or reproduce surveys without proper citation

Extremely worried

6. Given the symptoms you reported, did you attempt to receive a COVID-19 test?
   Yes
   No

If 6= Yes:

6.1. How many days passed between your first reported symptoms and your first attempt to receive a COVID-19 test?
   0 days
   1 day
   2 days
   3 days
   4 days
   5 days
   6 days
   7 days
   more than 7 days

6.2. How difficult was it to actually receive a COVID-19 test?
   Not at all difficult
   Slightly difficult
   Very difficult
   Extremely difficult

6.3. Did you receive a test for COVID-19 during the last two weeks in response to any symptoms reported above, any symptoms not reported, or for any other reason?
   Yes
   No

If 6.3 = Yes:

6.3.1. Where were you tested for COVID-19?

6.3.2. What was the result of your COVID-19 test?
   Result still pending
   Positive for COVID-19
   Negative for COVID-19
   Inconclusive result

Please provide the following information about your household contacts.

7. Are you currently living in your primary residence, or are you living in a temporary residence?
   Primary residence
   Temporary residence

If 17 = Primary residence, questions 7.1-7.2 display

Please do not reuse or reproduce surveys without proper citation

Appendix 3: Biweekly Survey for Healthcare Personnel

7.1. How many additional people (not including yourself) live or spend a significant amount of time (more than 40 hours per week on average) in your primary residence?

0
1
2
3
4
5
6
7
8
more

7.2. Has anyone in your primary residence had any symptoms (fever, cough, shortness of breath, fatigue, body aches, headache, diarrhea, sore throat, eye irritation, runny nose, changes in smell or taste, or a new rash) consistent with COVID-19 during the last two weeks?

Yes
No

If 7.2 =Yes:

7.2.1. How many people in your primary residence (not including you) have had symptoms consistent with COVID-19 during the last two weeks?

0
1
2
3
4
5
6
7
8
More

For each person identified in question 7.2.1, questions 7.2.2-7.2.6 repeat for each person.

7.2.2. What is your relationship to this person?

Partner or spouse
Child
Parent
Sibling
Other family member
In-home childcare provider or other caregiver
Other

If 7.2.2 = Other

7.2.2.1. Please specify your relationship with this person.

7.2.3. What is this person's age?

Please do not reuse or reproduce surveys without proper citation

7.2.4. Does this person also work in a healthcare facility?
- Yes
- No

If question 7.2.4 = Yes:

7.2.4.1. What is this person's position at their job in a healthcare facility?
- Physician
- Physician assistant
- Nurse practitioner
- Registered nurse
- Pharmacist
- Physical/occupational therapist
- Radiology technician
- Environmental services
- Food services
- Laboratory staff
- Other

7.2.4.2. Does this person work in any of the other high-risk facilities?
- Nursing home
- Prison
- Assisted living
- Other congregate residential facility
- Does not work in a high-risk facility

7.2.5. When did their symptoms consistent with COVID-19 first start?

7.2.6. Has this person been tested for COVID-19?
- Yes
- No

If 7.2.6 = Yes:

7.2.6.1. Where was this person tested for COVID-19?

7.2.6.2. What was the result of this person's COVID-19 test?
- Result still pending
- Positive for COVID-19
- Negative for COVID-19
- Inconclusive result

7.2.6.3. How difficult was it for this person to get tested for COVID-19?
- Not at all difficult
- slightly difficult
- very difficult
- extremely difficult

If 7 = Temporary residence, questions 7.3-7.4 display

Please do not reuse or reproduce surveys without proper citation

Appendix 3: Biweekly Survey for Healthcare Personnel

7.3. How many additional people (not including yourself) live or spend a significant amount of time (more than 40 hours per week on average) in your current temporary residence?

0
1
2
3
4
5
6
7
8
more

7.4. Has anyone in your current temporary residence had any symptoms (fever, cough, shortness of breath, fatigue, body aches, headache, diarrhea, sore throat, eye irritation, runny nose, changes in smell or taste, or a new rash) consistent with COVID-19 during the last two weeks? - Yes/No

If 7.4=Yes:

7.4.1. How many people in your current temporary residence (not including you) have had symptoms consistent with COVID-19 during the last two weeks?

0
1
2
3
4
5
6
7
8
more

For each person identified in question 7.4.1, questions 7.4.2-7.2.6 repeat for each person.

7.4.2. What is your relationship to this person?

Partner or spouse
Child
Parent
Sibling
Other family member
In-home childcare provider or other caregiver
Other

If 7.4.2 = Other

7.4.2.1. Please specify your relationship with this person.

7.4.3. What is this person's age?

7.4.4. Does this person also work in a healthcare facility?

Yes

Please do not reuse or reproduce surveys without proper citation

No

If 7.4,4 = Yes:

7.4.4.1. What is this person's position at their job in a healthcare facility?

Physician
Physician assistant
Nurse practitioner
Registered nurse
Pharmacist
Physical/occupational therapist
Radiology technician
Environmental services
Food services
Laboratory staff
Other

7.4.4.2. Does this person work in any of the other high-risk facilities?

Nursing home
Prison
Assisted living
Other congregate residential facility
Does not work in a high risk facility

7.4.5. When did their symptoms consistent with COVID-19 first start?

7.4.6. Has this person been tested for COVID-19? - Yes/No

If 7.4.6 = Yes:

7.4.6.1. Where was this person tested for COVID-19?

7.4.6.2. What was the result of this person's COVID-19 test?

Result still pending
Positive for COVID-19
Negative for COVID-19
Inconclusive result

7.4.6.3. How difficult was it for this person to get tested for COVID-19?

Not at all difficult
Slightly difficult
Very difficult
Extremely difficult

Please provide the general following information about PPE and sanitation.

8. How many times were you notified that you donned or doffed PPE incorrectly during the last two weeks?

0
1

Please do not reuse or reproduce surveys without proper citation

2
3
4
5
6
7
8
9
10 or more

If 8 > 0:
8.1. Please explain what happened when you donned/doffed PPE incorrectly.

9. How many times did you miss a hand or glove hygiene step during the PPE donning and doffing procedure during the last two weeks?
   0 times
   1 time
   2 times
   3 times
   4 times
   5 times
   6-10 times
   11-15 times
   16-20 times
   21 or more times

10. If you worked on a floor where the COVID patients are cohorted and PPE donning and doffing occurs at the entrance to the ward, how many times did you forget to perform hand or glove hygiene between patient rooms?
   0 times
   1 time
   2 times
   3 times
   4 times
   5 times
   6-10 times
   11-15 times
   16-20 times
   21 or more times
   NA (did not work on a ward with cohorted COVID patients)

11. How often have you worn a face mask in the community during the last two weeks?
   all of the time (100%)
   most of the time (75%)
   half of the time (50%)
   one-quarter of the time (25%)
   never (0%)

Please do not reuse or reproduce surveys without proper citation

Appendix 3: Biweekly Survey for Healthcare Personnel

12. How often have you worn a face mask at home during the last two weeks?
    - all of the time (100%)
    - most of the time (75%)
    - half of the time (50%)
    - one-quarter of the time (25%)
    - never (0%)

13. The questions in this scale ask you about your feelings and thoughts during the last month. Select Yes or No for each question
    - 13.1. I believed that my job was putting me at great risk
    - 13.2. I felt extra stress at work
    - 13.3. I was afraid of falling ill with COVID-19
    - 13.4. I felt I had little control over whether I would get infected or not
    - 13.5. I thought I would be unlikely to survive if I were to get COVID-19
    - 13.6. I thought about resigning because of COVID-19
    - 13.7. I was afraid I would pass COVID-19 on to others
    - 13.8. My family and friends were worried that they might get infected through me
    - 13.9. People avoided my family because of my work
    - 13.10. I was willing to accept the risks involved because I wanted to help the COVID-19 patients

14. The questions in this scale ask you about your feelings and thoughts during the last month. Select one for each question: never, almost never, sometimes, fairly often, very often
    - 14.1. In the last month, how often have you been upset because of something that happened unexpectedly?
    - 14.2. In the last month, how often have you felt that you were unable to control the important things in your life?
    - 14.3. In the last month, how often have you felt nervous and stressed?
    - 14.4. In the last month, how often have you felt confident about your ability to handle your personal problems?
    - 14.5. In the last month, how often have you felt that things were going your way?
    - 14.6. In the last month, how often have you felt that you were on top of things?
    - 14.7. In the last month, how often have you found that you could not cope with all of the things that you had to do?
    - 14.8. In the last month, how often have you been angered because of things that were outside of your control?
    - 14.9. In the last month, how often have you been able to control irritations in your life?
    - 14.10. In the last month, how often have you felt that difficulties were piling up so high that you could not overcome them?

15. In the last two weeks, how distressing were the following items? Select one for each question: Not distressing, somewhat distressing, moderately distressing, very distressing, extremely distressing
    - 15.1. Limited COVID-19 testing for healthcare personnel nationally
    - 15.2. Concerns about personal protective equipment nationally
    - 15.3. Lack of well-studied and effective COVID-19 medical treatments or vaccinations
    - 15.4. Lack of national clinical guidelines about post-discharge care for COVID-19 patients
    - 15.5. Potential transmission of COVID-19 from you to your family and loved ones

Please do not reuse or reproduce surveys without proper citation

15.6. The health and/or safety of your family and friends
15.7. Maintaining social distancing from your loved ones as a precautionary measure
15.8. Childcare/family care and/or home responsibilities
15.9. Personal financial planning, personal advance care planning
15.10. Contact with COVID-19 positive patients
15.11. Potential transmission of COVID-19 from the clinical environment to you
15.12. Potential transmission of COVID-19 from you to your colleagues
15.13. Potential transmission of COVID-19 from you to patients
15.14. Uncertain COVID-19 status of other healthcare personnel
15.15. Availability of personal protective equipment at work
15.16. Disturbed sleep (e.g., lack of sleep, insomnia, interrupted sleep, irregular sleep schedule)
15.17. Perceptions of a lack of control and/or uncertainty
15.18. Exposure to patient deaths
15.19. Difficult triage decisions or rationing of care (potential or actual) due to resource scarcity
15.20. Treating another healthcare personnel for suspected or confirmed COVID-19
15.21. Feelings of anxiety when working while having symptoms that may possibly be related to your own infection with COVID-19 (mild, unknown, or untested symptoms)
15.22. Performing clinical work that is outside of your specialty
15.23. Being redeployed or working outside of your expertise (but still within your specialty)
15.24. Number of hours worked
15.25. The need/expectation for healthcare personnel to provide social support (emotional labor) to patients due to lack of family visitation hours allowed for patients during this time

Please do not reuse or reproduce surveys without proper citation
