# Supplementary material for: SARS-CoV-2 Infection in Health Care Personnel and Their Household Contacts at a Tertiary Academic Medical Center: Protocol for a Longitudinal Cohort Study
Source: JMIR Res Protoc. 2021 Apr 30;10(4):e25410. doi: 10.2196/25410 (PMC8092024; doi:10.2196/25410)
Supplement: Multimedia Appendix 4 [file resprot_v10i4e25410_app4.pdf]

Appendix 4: Biweekly Survey for Healthcare Personnel (Weeks 12, 24, and 36 only)

1. Are you currently enrolled in any of the following scientific studies?
   - Not enrolled in any other studies
   - Enrolled in HERO trial of hydroxychloroquine
   - Enrolled in some other scientific study

   If 1 = Enrolled in some other scientific study:

   1.1. Please enter the name (or a brief description, if you don't know the name) of the other study you are enrolled in.

2. During the last two weeks, have you experienced any of the following symptoms? - Select Yes or No for each symptom
   2.1. fever (measured by thermometer or self-diagnosed)
   2.2. cough (new or worsening)
   2.3. shortness of breath (new or worsening)
   2.4. fatigue (new tiredness doing normal activities)
   2.5. body aches
   2.6. headache
   2.7. diarrhea
   2.8. sore throat
   2.9. itchy, pink, or painful eyes
   2.10. runny nose or congestion
   2.11. changes in your sense of smell or taste
   2.12. new rash
   2.13. repeated shaking with chills

If any of 2.1-2.13 are answered Yes, questions 3-6 display. If all of 2.1-2.13 are No, survey skips to question 7.

3. When did the symptoms reported above first start?

4. What did you do in response to the symptoms reported above?
   - Nothing
   - Took over the counter medication
   - Called Occupational Health
   - Visited Occupational Health
   - Called Respiratory Diagnostic Center
   - Visited Respiratory Diagnostic Center
   - Called outside clinic
   - Visited outside clinic
   - Other

   If 4 = Other:

   4.1. Please specify what other action you took in response to your symptoms.

5. Given the symptoms you reported, how worried were you that you may have been infected with COVID-19?

Please do not reuse or reproduce surveys without proper citation

Not at all worried
Slightly worried
Very worried
Extremely worried

6. Given the symptoms you reported, did you attempt to receive a COVID-19 test?
   Yes
   No

If 6= Yes:

6.1. How many days passed between your first reported symptoms and your first attempt to receive a COVID-19 test?
0 days
1 day
2 days
3 days
4 days
5 days
6 days
7 days
more than 7 days

6.2. How difficult was it to actually receive a COVID-19 test?
Not at all difficult
Slightly difficult
Very difficult
Extremely difficult

6.3. Did you receive a test for COVID-19 during the last two weeks in response to any symptoms reported above, any symptoms not reported, or for any other reason?
Yes
No

If 6.3 = Yes:

6.3.1. Where were you tested for COVID-19?

6.3.2. What was the result of your COVID-19 test?
Result still pending
Positive for COVID-19
Negative for COVID-19
Inconclusive result

Please provide the following information about your household contacts.

7. Are you currently living in your primary residence, or are you living in a temporary residence?

Please do not reuse or reproduce surveys without proper citation

Primary residence
Temporary residence

If 17 = Primary residence, questions 7.1-7.2 display

7.1. How many additional people (not including yourself) live or spend a significant amount of time (more than 40 hours per week on average) in your primary residence?
0
1
2
3
4
5
6
7
8 or more

7.2. Has anyone in your primary residence had any symptoms (fever, cough, shortness of breath, fatigue, body aches, headache, diarrhea, sore throat, eye irritation, runny nose, changes in smell or taste, or a new rash) consistent with COVID-19 during the last two weeks?
Yes
No

If 7.2 =Yes:

7.2.1. How many people in your primary residence (not including you) have had symptoms consistent with COVID-19 during the last two weeks?
0
1
2
3
4
5
6
7
8 or more

For each person identified in question 7.2.1, questions 7.2.2-7.2.6 repeat for each person.

7.2.2. What is your relationship to this person?
Partner or spouse
Child
Parent
Sibling
Other family member
In-home childcare provider or other caregiver
Other

Please do not reuse or reproduce surveys without proper citation

If 7.2.2 = Other

7.2.2.1. Please specify your relationship with this person.

7.2.3. What is this person's age?

7.2.4. Does this person also work in a healthcare facility?

Yes
No

If question 7.2.4 = Yes:

7.2.4.1. What is this person's position at their job in a healthcare facility?

Physician
Physician assistant
Nurse practitioner
Registered nurse
Pharmacist
Physical/occupational therapist
Radiology technician
Environmental services
Food services
Laboratory staff
Other

7.2.4.2. Does this person work in any of the other high-risk facilities?

Nursing home
Prison
Assisted living
Other congregate residential facility
Does not work in a high-risk facility

7.2.5. When did their symptoms consistent with COVID-19 first start?

7.2.6. Has this person been tested for COVID-19?

Yes
No

If 7.2.6 = Yes:

7.2.6.1. Where was this person tested for COVID-19?

7.2.6.2. What was the result of this person's COVID-19 test?

Result still pending
Positive for COVID-19
Negative for COVID-19
Inconclusive result

Please do not reuse or reproduce surveys without proper citation

7.2.6.3. How difficult was it for this person to get tested for COVID-19?
- Not at all difficult
- Slightly difficult
- Very difficult
- Extremely difficult

If 7 = Temporary residence, questions 7.3-7.4 display

7.3. How many additional people (not including yourself) live or spend a significant amount of time (more than 40 hours per week on average) in your current temporary residence?
- 0
- 1
- 2
- 3
- 4
- 5
- 6
- 7
- 8 or more

7.4. Has anyone in your current temporary residence had any symptoms (fever, cough, shortness of breath, fatigue, body aches, headache, diarrhea, sore throat, eye irritation, runny nose, changes in smell or taste, or a new rash) consistent with COVID-19 during the last two weeks?
- Yes
- No

If 7.4=Yes:

7.4.1. How many people in your current temporary residence (not including you) have had symptoms consistent with COVID-19 during the last two weeks?
- 0
- 1
- 2
- 3
- 4
- 5
- 6
- 7
- 8 or more

For each person identified in question 7.4.1, questions 7.4.2-7.2.6 repeat for each person.

7.4.2. What is your relationship to this person?
- Partner or spouse
- Child
- Parent
- Sibling

Please do not reuse or reproduce surveys without proper citation

Other family member
In-home childcare provider or other caregiver
Other

If 7.4.2 = Other
7.4.2.1. Please specify your relationship with this person.

7.4.3. What is this person's age?

7.4.4. Does this person also work in a healthcare facility?
Yes
No

If 7.4,4 = Yes:
7.4.4.1. What is this person's position at their job in a healthcare facility?
Physician
Physician assistant
Nurse practitioner
Registered nurse
Pharmacist
Physical/occupational therapist
Radiology technician
Environmental services
Food services
Laboratory staff
Other

7.4.4.2. Does this person work in any of the other high-risk facilities?
Nursing home
Prison
Assisted living
Other congregate residential facility
Does not work in a high risk facility

7.4.5. When did their symptoms consistent with COVID-19 first start?

7.4.6. Has this person been tested for COVID-19?
Yes
No

If 7.4.6 = Yes:
7.4.6.1. Where was this person tested for COVID-19?

7.4.6.2. What was the result of this person's COVID-19 test?
Result still pending

Please do not reuse or reproduce surveys without proper citation

Positive for COVID-19
Negative for COVID-19
Inconclusive result

7.4.6.3. How difficult was it for this person to get tested for COVID-19?
Not at all difficult
Slightly difficult
Very difficult
Extremely difficult

Please provide the general following information about PPE and sanitation.

8. How many times were you notified that you donned or doffed PPE incorrectly during the last two weeks?
   0
   1
   2
   3
   4
   5
   6
   7
   8
   9
   10 or more

   If 8 > 0:
   8.1. Please explain what happened when you donned/doffed PPE incorrectly.

9. How many times did you miss a hand or glove hygiene step during the PPE donning and doffing procedure during the last two weeks?
   0 times
   1 time
   2 times
   3 times
   4 times
   5 times
   6-10 times
   11-15 times
   16-20 times
   21 or more times

10. If you worked on a floor where the COVID patients are cohorted and PPE donning and doffing occurs at the entrance to the ward, how many times did you forget to perform hand or glove hygiene between patient rooms?

Please do not reuse or reproduce surveys without proper citation

0 times
1 time
2 times
3 times
4 times
5 times
6-10 times
11-15 times
16-20 times
21 or more times
NA (did not work on a ward with cohorted COVID patients)

11. How often have you worn a face mask in the community during the last two weeks?
    all of the time (100%)
    most of the time (75%)
    half of the time (50%)
    one-quarter of the time (25%)
    never (0%)

12. How often have you worn a face mask at home during the last two weeks?
    all of the time (100%)
    most of the time (75%)
    half of the time (50%)
    one-quarter of the time (25%)
    never (0%)

13. To what extent do you agree that wearing a face mask could prevent contracting and spreading the novel coronavirus?
    Not at all
    To a very small extent
    To a moderate extent
    To a very great extent

14. Do you think you have adequate knowledge about COVID-19?
    Not at all
    To a very small extent
    To a moderate extent
    To a very great extent

15. To what extent do you believe you are able to wear a face mask properly?
    Not at all
    To a very small extent
    To a moderate extent
    To a very great extent

Please do not reuse or reproduce surveys without proper citation

Appendix 4: Biweekly Survey for Healthcare Personnel (Weeks 12, 24, and 36 only)

16. Do you think that COVID-19 will be successfully controlled?
    - Yes
    - No
    - Not sure

17. If you have practiced extended use of an N95 respirator at work, how confident are you that your extended use respirator will protect you from COVID-19?
    - Not confident at all
    - Only slightly confident
    - Somewhat confident
    - Moderately confident
    - Very confident
    - Extremely confident
    - Not applicable

18. During the last two weeks, have you experienced the following due to the COVID-19 pandemic? Select one for each question: Yes/No

    18.1. I believed that my job was putting me at great risk
    18.2. I felt extra stress at work
    18.3. I was afraid of falling ill with COVID-19
    18.4. I felt I had little control over whether I would get infected or not
    18.5. I thought I would be unlikely to survive if I were to get COVID-19
    18.6. I thought about resigning because of COVID-19
    18.7. I was afraid I would pass COVID-19 on to others
    18.8. My family and friends were worried that they might get infected through me
    18.9. People avoided my family because of my work
    18.10. I was willing to accept the risks involved because I wanted to help the COVID-19 patients

19. The questions in this scale ask you about your feelings and thoughts during the last month. Select one for each question: never, almost never, sometimes, fairly often, very often

    19.1. In the last month, how often have you been upset because of something that happened unexpectedly?
    19.2. In the last month, how often have you felt that you were unable to control the important things in your life?
    19.3. In the last month, how often have you felt nervous and stressed?
    19.4. In the last month, how often have you felt confident about your ability to handle your personal problems?
    19.5. In the last month, how often have you felt that things were going your way?
    19.6. In the last month, how often have you found that you could not cope with all of the things that you had to do?
    19.7. In the last month, how often have you been able to control irritations in your life?
    19.8. In the last month, how often have you felt that you were on top of things?

Please do not reuse or reproduce surveys without proper citation

19.9. In the last month, how often have you been angered because of things that were outside of your control?
19.10. In the last month, how often have you felt that difficulties were piling up so high that you could not overcome them?

20. In the last two weeks, how distressing were the following items? Select one for each question: Not distressing, somewhat distressing, moderately distressing, very distressing, extremely distressing

20.1. Limited COVID-19 testing for healthcare personnel nationally
20.2. Concerns about personal protective equipment nationally
20.3. Lack of well-studied and effective COVID-19 medical treatments or vaccinations
20.4. Lack of national clinical guidelines about post-discharge care for COVID-19 patients

21. In the last two weeks, how distressing were the following items? Select one for each question: Not distressing, somewhat distressing, moderately distressing, very distressing, extremely distressing
21.1. Potential transmission of COVID-19 from you to your family and loved ones
21.2. The health and/or safety of your family and friends
21.3. Maintaining social distancing from your loved ones as a precautionary measure
21.4. Childcare/family care and/or home responsibilities
21.5. Personal financial planning, personal advance care planning

22. In the last two weeks, how distressing were the following items? Select one for each question: Not distressing, somewhat distressing, moderately distressing, very distressing, extremely distressing
22.1. Contact with COVID-19 positive patients
22.2. Potential transmission of COVID-19 from the clinical environment to you
22.3. Potential transmission of COVID-19 from you to your colleagues
22.4. Potential transmission of COVID-19 from you to patients
22.5. Uncertain COVID-19 status of other healthcare personnel
22.6. Availability of personal protective equipment at work
22.7. Disturbed sleep (e.g., lack of sleep, insomnia, interrupted sleep, irregular sleep schedule)
22.8. Perceptions of a lack of control and/or uncertainty
22.9. Exposure to patient deaths
22.10. Difficult triage decisions or rationing of care (potential or actual) due to resource scarcity
22.11. Treating another healthcare personnel for suspected or confirmed COVID-19
22.12. Feelings of anxiety when working while having symptoms that may possibly be related to your own infection with COVID-19 (mild, unknown, or untested symptoms)
22.13. Performing clinical work that is outside of your specialty
22.14. Being redeployed or working outside of your expertise (but still within your specialty)
22.15. Number of hours worked
22.16. The need/expectation for healthcare personnel to provide social support (emotional labor) to patients due to lack of family visitation hours allowed for patients during this time

23. During the last two weeks, how often have you been bothered by the following problems? Select one for each problem: not at all, several days, more than half of the days, nearly every day
23.1. Feeling nervous, anxious, or on edge

Please do not use or reproduce surveys without proper citation

23.2. Not being able to stop or control worrying
23.3. Worrying too much about different things
23.4. Trouble relaxing
23.5. Being so restless that it's hard to sit still
23.6. Becoming easily annoyed or irritable
23.7. Feeling afraid as if something awful might happen

If any from 23.1-23.7 is NOT "not at all":

23.7.1. How difficult have these problems made it for you to do your work, take care of things at home, or get along with other people?

not difficult
somewhat difficult
very difficult
extremely difficult

24. During the last two weeks, how often have you been bothered by the following problems? Select one for each item: not at all, several days, more than half of the days, nearly every day

24.1. Little interest or pleasure in doing things
24.2. Feeling down, depressed, or hopeless
24.3. Trouble falling or staying asleep, or sleeping too much
24.4. Feeling tired or having little energy
24.5. Poor appetite or overeating
24.6. Feeling bad about yourself, or that you are a failure or have let yourself or your family down
24.7. Trouble concentrating on things, such as reading the newpaper or watching television
24.8. Moving or speaking so slowly that other people could have noticed, or the opposite - being so fidgety or restless that you have been moving around a lot more than usual
24.9. Thoughts that you would be better off dead, or of hurting yourself

If any question from 24.1-24..9 is NOT "not at all":

24.9.1. How difficult have these problems made it for you to do your work, take care of things at home, or get along with other people?

not difficult
somewhat difficult
very difficult
extremely difficult

25. In your life, have you ever had any experience that was so frightening, horrible, or upsetting that, in the past month, you: (Select Yes or No for each item)

25.1. Have had nightmares about it or thought about it when you did not want to
25.2. Tried hard not to think about it or went out of your way to avoid situations that reminded you of it
25.3. Were constantly on guard, watchful, or easily startled
25.4. Felt numb or detached from others, activities, or your surroundings

Please do not reuse or reproduce surveys without proper citation
