# Supplementary material for: SARS-CoV-2 Infection in Health Care Personnel and Their Household Contacts at a Tertiary Academic Medical Center: Protocol for a Longitudinal Cohort Study
Source: JMIR Res Protoc. 2021 Apr 30;10(4):e25410. doi: 10.2196/25410 (PMC8092024; doi:10.2196/25410)
Supplement: Multimedia Appendix 6 [file resprot_v10i4e25410_app6.pdf]

Appendix 6: Baseline Survey for Household Participants

Please provide the following personal and health information.

1. How are you related to the primary study participant?
   I am the participant's partner or spouse
   I am the participant's child
   I am the participant's parent
   I am the participant's sibling
   I am the participant's cousin, uncle, aunt, grandparent, etc.
   I am the participant's non-related housemate

2. What is your sex?
   Female
   Male

3. What is your race? *Select all that apply*
   American Indian or Alaska Native
   Asian
   Black or African American
   Native Hawaiian or Pacific Islander
   White
   Other

   If 3 = Other:
   3.1. By what other race do you identify?

4. What is your ethnicity?
   Hispanic or Latinx
   Not Hispanic or Latinx
   Other

5. What is the highest level of education or schooling you have completed?
   Never attended school
   Kindergarten - 8th grade
   Some high school
   High school equivalency (GED)
   High school graduate
   Some college
   College graduate
   Graduate school

6. Before the COVID-19 pandemic began in North Carolina, which of the following best described your work situation?
   Worked full time
   Worked part time

Please do not reuse or reproduce surveys without proper citation

Was looking for work/employment
Retired, homemaker
Student
On maternity/paternity leave
On illness/sick leave
On disability
Other

7. Before the COVID-19 pandemic began in North Carolina, did you consider yourself self-employed (including as as an independent contractor or gig-economy worker)? - Yes/No

8. Of the job (or jobs) that you held before the COVID-19 pandemic in North Carolina, which description best fits your main job (i.e. the job you spent the most hours at, or the job at which you had worked the longest)?
   Managerial
   Professional
   Administrative support
   Service
   Farming, forestry, or fishing
   Precision production, craft, or repair
   Operators, fabricators, or laborers
   Military
   Student
   Not working
   Other

9. In your main job before the COVID-19 pandemic, how often were you required to work from outside of the home?
   Always
   Often
   Sometimes
   Hardly ever
   Never

10. Has your work situation changed since the COVID-19 pandemic began in North Carolina? - Yes/no

If 10 = Yes:
10.1. Which of the following best describes your current work situation?
   Worked full time
   Worked part time
   Was looking for work/employment
   Retired
   Homemaker

Please do not reuse or reproduce surveys without proper citation

Student
On maternity/paternity leave
On illness/sick leave
On disability
Other

10.2. Do you consider yourself self-employed (including as an independent contractor or gig-economy worker)?
Yes
No

10.3. Which description best fits your current main job (i.e. the job you spend the most hours at, or the job at which you have worked the longest)?
Managerial
Professional
Administrative support
Service
Farming, forestry, or fishing
Precision production, craft, or repair
Operators, fabricators, or laborers
Military
Student
Not working
Other

10.4. How often are you required to work from outside of the home?
Always
Often
Sometimes
Hardly ever
Never

11. Have you worked in health care or in a health care facility (i.e. hospital, clinic, urgent care, residential care facility, etc.) since the beginning of the COVID-19 pandemic in North Carolina?
Yes
No

If 11 = Yes:
11.1. What is your role in health care?
Physician
Physician assistant
Nurse practitioner
Registered nurse

Please do not reuse or reproduce surveys without proper citation

Pharmacist
Physical/occupational therapist
Radiology technician
Environmental services
Food services
Laboratory staff
Other

11.2. How many hours per week are you currently working in health care in person (i.e. excluding telehealth hours)?
0
1-10
11-20
21-40
41-60
61-80
81 or more

12. Do you currently smoke cigarettes, cigars, or a pipe on a daily basis?
Yes
No

If 12 = Yes:
12.1. How old were you when you first started to smoke fairly regularly?

12.2. What is the average number of cigarettes smoked per day since you began smoking?
None
1-10
11-20
21-30
31-40
41-50
51-60
61 or more

12.3. What is the average number of cigars smoked per day since you began smoking?
None
1
2
3
4
5 or more

Please do not reuse or reproduce surveys without proper citation

12.4. What is the average number of bowls of tobacco smoked per day since you began smoking?

None
1
2
3
4
5 or more

13. Did you previously smoke cigarettes, cigars, or a pipe on a daily basis?

Yes
No

If 13 = Yes:

13.1. How old were you when you first started to smoke fairly regularly?

13.2. How many years has it been since you quit smoking?

13.3. What was the average number of cigarettes smoked per day when you were smoking?

None
1-10
11-20
21-30
31-40
41-50
51-60
61 or more

13.4. What was the average number of cigarettes smoked per day when you were smoking?

None
1-10
11-20
21-30
31-40
41-50
51-60
61 or more

13.5. What was the average number of cigars smoked per day when you were smoking?

None
1
2
3

Please do not reuse or reproduce surveys without proper citation

4
5 or more

13.6. What was the average number of bowls of tobacco smoked per day when you were smoking?

None
1
2
3
4
5 or more

14. Do you currently use electronic cigarettes (e-cigarettes,vaping)?

Yes
No

If 14 = Yes:

14.1. How old were you when you first started to use electronic cigarettes fairly regularly?

14.2. What is the average number of e-cigarette (or other vaping product) puffs did you inhale per day?

0-25
26-50
51-75
76-100
101-125
126-150
151-175
176-200
201-225
226-250
251 or more

If 14 = No:

14.3. Did you previously use electronic cigarettes (e-cigarettes, vaping)?

Yes
No

If 14.3 = Yes:

14.3.1. How old were you when you first started to use electronic cigarettes fairly regularly?

14.3.2. How many years has it been since you quit using electronic cigarettes?

Please do not reuse or reproduce surveys without proper citation

14.3.3. What was the average number of e-cigarette (or other vaping product) puffs did you inhale per day?

0-25
26-50
51-75
76-100
101-125
126-150
151-175
176-200
201-225
226-250
251 or more

15. Do you currently drink alcohol at least once a week?

Yes
No

If 15 = Yes:

15.1. How old were you when you first started to drink alcohol fairly regularly?

15.2. On how many weekdays (Monday through Friday) do you usually drink alcohol?

0
Only occasionally
1
2
3
4
5

If 15.2 > 0:

15.2.1. When you drink on a weekday (Monday through Friday), how many drinks do you usually have? (One drink is equal to 5 ounces of wine, 12 ounces of beer, or 1.5 ounces of liquor)

1
2
3
4
5
6
7 or more

Please do not reuse or reproduce surveys without proper citation

15.3. On how many weekend days (Saturday and Sunday) do you usually drink alcohol?

0
Only occasionally
1
2

If 15.3 > 0:

15.3.1. When you drink on a weekend day (Saturday and Sunday), how many drinks do you usually have? (One drink is equal to 5 ounces of wine, 12 ounces of beer, or 1.5 ounces of liquor)

1
2
3
4
5
6
7 or more

If 15 = No:

15.4. Did you previously drink alcohol at least once a week?

If 15.4 = Yes:

15.4.1. How old were you when you first started to drink alcohol fairly regularly?

15.4.2. How many years has it been since you quit drinking alcohol?

15.4.3. On how many weekdays (Monday through Friday) did you usually drink alcohol?

0
Only occasionally
1
2
3
4
5

If 15.4.3 > 0:

15.4.3.1. When you drank on a weekday (Monday through Friday), how many drinks did you usually have? (One drink is equal to 5 ounces of wine, 12 ounces of beer, or 1.5 ounces of liquor)

1
2
3

Please do not reuse or reproduce surveys without proper citation

4
5
6
7 or more

15.4.4. On how many weekend days (Saturday and Sunday) did you usually drink alcohol?
0
Only occasionally
1
2

If 15.4.4 >0,

15.4.4.1. When you drank on a weekend day (Saturday and Sunday), how many drinks did you usually have? (One drink is equal to 5 ounces of wine, 12 ounces of beer, or 1.5 ounces of liquor)
1
2
3
4
5
6
7 or more

16. At least once a week, do you engage in regular activity like brisk walking, jogging, bicycling, swimming, etc. long enough to work up a sweat, get your heart thumping, or get out of breath?
Yes
No

If 16 = Yes:

16.1. On average, how many days per week do you engage in this kind of exercise?
1
2
3
4
5
6
7

16.2. On average, how many minutes per day do you engage in this kind of exercise?
0-20
21-40
41-60

Please do not reuse or reproduce surveys without proper citation

61 or more

16.3. When you are exercising in your usual fashion, how would you rate your average level of exertion (degree of effort)?

Easy / Warm-up
Medium (can hold a conversation) / Aerobic Development
Hard (but you can push yourself to continue) / Aerobic Endurance
Very Hard (cannot hold a conversation) / Anaerobic Endurance
Extremely Hard (out of breath, your body wants to stop) / Speed, Power

17. Have you ever received a diagnosis of any of the following? Select Yes or No for each diagnosis

17.1. Seasonal allergies
17.2. Asthma
17.3. Diabetes
17.4. Hypertension
17.5. Cardiovascular disease
17.6. Cancer
17.7. Chronic lung or respiratory disease
17.8. Other chronic condition

If 17.8 = Yes:

17.8.1. Please identify what other chronic medical condition you have been diagnosed with.

18. Have you previously been diagnosed with COVID-19?

Yes
No

If 18 = Yes:

18.1. When were you previously diagnosed with COVID-19? Please provide your best guess as to your date of testing, or if not tested for COVID-1 then your best guess as to the date when you were diagnosed by a clinician.

18.2. Please provide your best guess of how you were exposed to and infected with COVID-19

Travel (airport, bus station, etc.)
Community transmission (retail setting, etc.)
Household contact (sick household or family member, etc.)
Occupational (clinic or hospital exposure, etc.)
Other
Unknown

If 18.2=Other,

18.2.1. Please explain what other route of exposure you experienced.

Please do not reuse or reproduce surveys without proper citation

If 18 = No:

18.3. Whether or not you were tested and diagnosed, have you previously experienced symptoms that made you believe you were infected with COVID-19?

Yes
No

If 18.3 = Yes:

18.3.1. When did you first experience symptoms that made you believe you were infected with COVID-19? Please provide your best guess of the date when you first believed that you may have had COVID-19.

18.3.2. Please provide your best guess of how you may have been exposed to, and infected with, COVID-19.

Travel (airport, bus station, etc.)
Community transmission (retail setting, etc.)
Household contact (sick household or family member, etc.)
Occupational (clinic or hospital exposure, etc.)
Other
Unknown

If 18.3.2 = Other:

18.3.2.1. Please explain what other route of exposure you experienced.

19. During the last two weeks, have you experienced any of the following symptoms? Select Yes or No for each symptom

19.1. fever (measured by thermometer or self-diagnosed)
19.2. cough (new or worsening)
19.3. shortness of breath (new or worsening)
19.4. fatigue (new tiredness doing normal activities)
19.5. body aches
19.6. headache
19.7. diarrhea
19.8. sore throat
19.9. itchy, pink, or painful eyes
19.10. runny nose or congestion
19.11. changes in your sense of smell or taste
19.12. new rash
19.13. repeated shaking with chills

If any of the questions 19.1-19.13 =Yes, questions 20-23 display. If none of 19.1 to 19.13=Yes, survey skips to question 24.

20. When did the symptoms reported above first start?

Please do not reuse or reproduce surveys without proper citation

Appendix 6: Baseline Survey for Household Participants

21. What did you do in response to the symptoms reported above?
    - Nothing
    - took over the counter medication
    - called Occupational Health
    - visited Occupational Health
    - called Respiratory Diagnostic Center
    - visited Respiratory Diagnostic Center
    - called outside clinic; visited outside clinic
    - Other

    If 21 = Other,

    21.1. Please specify what other action you took in response to your symptoms.

22. Given the symptoms you reported, how worried were you that you may have been infected with COVID-19?
    - not at all worried
    - slightly worried
    - very worried
    - extremely worried

23. Given the symptoms you reported, did you attempt to receive a COVID-19 test?
    - Yes
    - No

    If 23 = Yes:

    23.1. How many days passed between your first reported symptoms and your first attempt to receive a COVID-19 test?
    - 0 days
    - 1 day
    - 2 days
    - 3 days
    - 4 days
    - 5 days
    - 6 days
    - 7 days
    - more than 7 days

    23.2. How difficult was it to actually receive a COVID-19 test?
    - not at all difficult
    - slightly difficult
    - very difficult
    - extremely difficult

Please do not reuse or reproduce surveys without proper citation

24. Did you receive a test for COVID-19 during the last two weeks in response to the symptoms you reported above or for any other reason not reported?

    Yes
    No

    If 24 = Yes:

    24.1. Where were you tested for COVID-19?

    24.2. What was the result of your COVID-19 test?

    Result still pending
    Positive for COVID-19
    Negative for COVID-19
    Inconclusive result

25. To what extent do you agree that wearing a face mask could prevent contracting and spreading the novel coronavirus?

    Not at all
    To a very small extent
    To a moderate extent
    To a very great extent

26. Do you think you have adequate knowledge about COVID-19?

    Not at all
    To a very small extent
    To a moderate extent
    To a very great extent

27. To what extent do you believe you are able to wear a face mask properly?

    Not at all
    To a very small extent
    To a moderate extent
    To a very great extent

28. Do you think that COVID-19 will be successfully controlled?

    Yes
    No
    Not sure

Please do not reuse or reproduce surveys without proper citation
