# Supplementary material for: SARS-CoV-2 Infection in Health Care Personnel and Their Household Contacts at a Tertiary Academic Medical Center: Protocol for a Longitudinal Cohort Study
Source: JMIR Res Protoc. 2021 Apr 30;10(4):e25410. doi: 10.2196/25410 (PMC8092024; doi:10.2196/25410)
Supplement: Multimedia Appendix 7 [file resprot_v10i4e25410_app7.pdf]

Appendix 7: Weekly Survey for Household Participants

1. What is your first name?

2. What is your last name?

3. During the last week, have you experienced any of the following symptoms? Select Yes or No for each symptom.
   - 3.1. fever (measured by thermometer or self-diagnosed)
   - 3.2. cough (new or worsening)
   - 3.3. shortness of breath (new or worsening)
   - 3.4. fatigue (new tiredness doing normal activities)
   - 3.5. body aches
   - 3.6. headache
   - 3.7. diarrhea
   - 3.8. sore throat
   - 3.9. itchy, pink, or painful eyes
   - 3.10. runny nose or congestion
   - 3.11. changes in your sense of smell or taste
   - 3.12. new rash
   - 3.13. repeated shaking with chills

4. Did you receive a test for COVID-19 during the last week in response to the symptoms you reported above or for any other reason not reported?

   Yes
   No

   If 4 = Yes:

   4.1. Where were you tested for COVID-19?

   4.2. What was the result of your COVID-19 test?

   Result still pending
   Positive for COVID-19
   Negative for COVID-19
   Inconclusive result

Please do not reuse or reproduce surveys without proper citation
