# Supplementary material for: SARS-CoV-2 Infection in Health Care Personnel and Their Household Contacts at a Tertiary Academic Medical Center: Protocol for a Longitudinal Cohort Study
Source: JMIR Res Protoc. 2021 Apr 30;10(4):e25410. doi: 10.2196/25410 (PMC8092024; doi:10.2196/25410)
Supplement: Multimedia Appendix 8 [file resprot_v10i4e25410_app8.pdf]

Appendix 8. Day 21 Survey for Household Participants

Please provide the following personal and health information.

1. How are you related to the primary study participant?
   - I am the participant's partner or spouse
   - I am the participant's child
   - I am the participant's parent
   - I am the participant's sibling
   - I am the participant's cousin, uncle, aunt, grandparent, etc.
   - I am the participant's non-related housemate

2. Before the COVID-19 pandemic began in North Carolina, which of the following best described your work situation?
   - Worked full time
   - Worked part time
   - Was looking for work/employment
   - Retired or homemaker
   - Student
   - On maternity/paternity leave
   - On illness/sick leave
   - On disability
   - Other

3. Before the COVID-19 pandemic began in North Carolina, did you consider yourself self-employed (including as an independent contractor or gig-economy worker)? - Yes/No

4. Of the job (or jobs) that you held before the COVID-19 pandemic in North Carolina, which description best fits your main job (i.e. the job you spent the most hours at, or the job at which you had worked the longest)?
   - Managerial
   - Professional
   - Administrative support
   - Service
   - Farming, forestry, or fishing
   - Precision production
   - Craft or repair
   - Operators
   - Fabricators, or laborers
   - Military
   - Student
   - Not working
   - Other

5. In your main job before the COVID-19 pandemic, how often were you required to work from outside of the home?
   - Always
   - Often
   - Sometimes
   - Hardly ever

Please do not reuse or reproduce surveys without proper citation

Never

6. Has your work situation changed since the COVID-19 pandemic began in North Carolina?
   Yes
   No

If 6 = Yes:

6.1. Which of the following best describes your current work situation?
   Worked full time
   Worked part time
   Was looking for work/employment
   Retired or homemaker
   Student
   On maternity/paternity leave
   On illness/sick leave
   On disability
   Other

6.2. Do you consider yourself self-employed (including as an independent contractor or gig-economy worker)?
   Yes
   No

6.3. Which description best fits your current main job (i.e. the job you spend the most hours at, or the job at which you have worked the longest)?
   Managerial
   Professional
   Administrative support
   Service
   Farming, forestry, or fishing
   Precision production
   Craft or repair
   Operators
   Fabricators, or laborers
   Military
   Student
   Not working
   Other

6.4. How often are you required to work from outside of the home?
   Always
   Often
   Sometimes
   Hardly ever
   Never

6.5. Have you worked in health care or in a health care facility (i.e. hospital, clinic, urgent care, residential care facility, etc.) since the beginning of the COVID-19 pandemic in North Carolina? -
   Yes

Please do not reuse or reproduce surveys without proper citation

Appendix 8. Day 21 Survey for Household Participants

No

If 6.5 = Yes:

6.5.1. What is your role in health care?

Physician
Physician assistant
Nurse practitioner
Rregistered nurse
Pharmacist
Physical/occupational therapist
Radiology technician
Environmental services
Food services
Laboratory staff
Other

6.5.2. How many hours per week are you currently working in health care in person (i.e. excluding telehealth hours)?

0
1-10
11-20
21-40
41-60
61-80
81 or more

7. Have you been diagnosed with COVID-19 since your last survey?

Yes
No

If 7 = Yes:

7.1. When were you diagnosed with COVID-19?

7.2. Please provide your best guess of how you were exposed to, and infected with, COVID-19.

Travel (airport, bus station, etc.)
Community transmission (retail setting, etc.)
Household contact (sick household or family member, etc.)
Occupational (clinic or hospital exposure, etc.)
Other
Unknown

If 7.2 = Other:

7.2.1. Please explain what other route of exposure you experienced.

If 7 =No,

7.3. Whether or not you were tested and diagnosed, have you experienced symptoms that made you believe you were infected with COVID-19 since your last survey?

Please do not reuse or reproduce surveys without proper citation

Appendix 8. Day 21 Survey for Household Participants

Yes
No

If 7.3 = Yes:

7.3.1. When did you first experience symptoms that made you believe you were infected with COVID-19?

7.3.2. Please provide your best guess of how you may have been exposed to, and infected with, COVID-19

Travel (airport, bus station, etc.)
Community transmission (retail setting, etc.)
Household contact (sick household or family member, etc.)
Occupational (clinic or hospital exposure, etc.)
Other
Unknown

If 7.3.2. = Other

7.3.2.1. Please explain what other route of exposure you experienced.

8. Have you experienced any of the following symptoms since your last survey? Select Yes or No for each symptom.
   8.1. fever (measured by thermometer or self-diagnosed)
   8.2. cough (new or worsening)
   8.3. shortness of breath (new or worsening)
   8.4. fatigue (new tiredness doing normal activities)
   8.5. body aches
   8.6. headache
   8.7. diarrhea
   8.8. sore throat
   8.9. itchy, pink, or painful eyes
   8.10. runny nose or congestion
   8.11. changes in your sense of smell or taste
   8.12. new rash
   8.13. repeated shaking with chills

If any of questions 8.1 – 8.13 =Yes, questions 9-13 display. If none of questions 8.1-8.13 = Yes, survey skips ahead to question 14.

9. When did the symptoms reported above first start?

10. What did you do in response to the symptoms reported above?
    10.1. Nothing
    10.2. took over the counter medication
    10.3. called Occupational Health
    10.4. visited Occupational Health
    10.5. called Respiratory Diagnostic Center
    10.6. visited Respiratory Diagnostic Center
    10.7. called outside clinic

Please do not reuse or reproduce surveys without proper citation

Appendix 8. Day 21 Survey for Household Participants

10.8. visited outside clinic
10.9. other

If 10.9 = other:
10.9.1. Please specify what other action you took in response to your symptoms.

11. Given the symptoms you reported, how worried were you that you may have been infected with COVID-19?
    - Not at all worried
    - Slightly worried
    - Very worried
    - Extremely worried

12. Given the symptoms you reported, how worried were you that you may have been infected with COVID-19?
    - Not at all worried
    - Slightly worried
    - Very worried
    - Extremely worried

13. Given the symptoms you reported, did you attempt to receive a COVID-19 test?
    - Yes
    - No

If 13 = Yes:
13.1. How many days passed between your first reported symptoms and your first attempt to receive a COVID-19 test?
- 0 days
- 1 day
- 2 days
- 3 days
- 4 days
- 5 days
- 6 days
- 7 days
- more than 7 days

13.2. How difficult was it to actually receive a COVID-19 test?
- Not at all difficult
- Slightly difficult
- Very difficult
- Extremely difficult

14. Did you receive a test for COVID-19 during the last two weeks in response to the symptoms you reported above or for any other reason not reported?
    - Yes
    - No

Please do not reuse or reproduce surveys without proper citation

Appendix 8. Day 21 Survey for Household Participants

If 14 =Yes:

14.1. Where were you tested for COVID-19?

14.2. What was the result of your COVID-19 test?

Result still pending
Positive for COVID-19
Negative for COVID-19
Inconclusive result

Please do not reuse or reproduce surveys without proper citation
