# Supplementary material for: SARS-CoV-2 Infection in Health Care Personnel and Their Household Contacts at a Tertiary Academic Medical Center: Protocol for a Longitudinal Cohort Study
Source: JMIR Res Protoc. 2021 Apr 30;10(4):e25410. doi: 10.2196/25410 (PMC8092024; doi:10.2196/25410)
Supplement: Multimedia Appendix 9 [file resprot_v10i4e25410_app9.pdf]

COVID HCP Study Laboratory Protocol

Table of Contents

Sample Collection Details and Laboratory Preparation **1**

Solutions for Sample Processing **2**

Processing of Mid-Turbinate Nasal Swab (MTNS) **3**

RNA Extraction 3

PCR Protocol 3

Processing of Blood Tubes **6**

Processing of Tasso Serum Sample **10**

Enzyme-linked immunosorbent assay (ELISA) Protocol **11**

Sample Collection Details and Laboratory Preparation

Protocol based on collection of 1 X EDTA tube, 2 X Ficoll tube, and 1 X mid-turbinate nasal swab (MTNS). *For all visits except Week 0 and Week 12, only 1 X Ficoll tube will be collected.* A Tasso serum separator tube (SST) may also be collected if participants opt into that sub-study. Study staff will leave Ficoll (and Tasso SST, if collected) tubes at room temperature in a BSL2 biosafety cabinet (BSC) and EDTA tubes and MTNS samples at 4°C.

Peripheral blood mononuclear cells (PBMCs) to be isolated and stored only on samples from baseline visit and week 12 visit or if rapid diagnostic test (RDT) is positive for IgM or IgG.

Before Starting:

- Turn on water bath to 56°C
- Prepare 10% bleach solution in waste bucket, place waste bucket inside hood
- Print labels based on guideline below

At the beginning of each day of processing, use the manifest of visits and chain of custody forms that day to print labels for sample tubes

- If no PBMCs will be collected, you will need 7 labels
- If PBMCs will be collected, you will need 13 labels

Labels should include:

- Sample ID#
- Date of collection
- Sample type (S- serum, SW- swab, PBMC- PBMC cells, RDT- rapid diagnostic test, BSP- blood spot paper, SST- Tasso sub-study)

Ensure all steps are carried out in proper biosafety level BSC unless indicated. All staff must don personal protective equipment (PPE) per their institutional protocols. All spills to be cleaned per laboratory protocol. Any exposure to biologic material should be reported to Occupational Health and the

Solutions for Sample Processing

Use an autoclaved, sterile glass bottle for making the amount you will need for the week.Use aseptic technique and sterile pipettes when making reagents.Discard and remake the reagents after one week.Store regents at 4°C for the week.

PBMC Freezing Medium 1 (prepare a master mix based on these per participant volumes)

- 1ml FBS (Life technologies 10082147)
- 1.5ml RPMI 1640 (Invitrogen 11875119)
- Store at 4°C for up to one week.

PBMC Freezing Medium 2 (prepare a master mix based on these per participant volumes)

- 0.5ml DMSO (Sigma D8418-500ml)
- 2ml RPMI 1640
- Store at 4°C for up to one week.

Processing of Mid-Turbinate Nasal Swab (MTNS)

Equipment needed:
- Sample in BSC
- 2 inch high freezer safe storage box
- 1ml pipette
- 2 X 2ml O-ring rubes (2mL cryo vial, freezer safe and sterile)
- 2 printed labels for each sample
- Pipettor
- Vortex
- Racks for tubes
- QIAamp® 96 Virus QIAcube® HT Kit

Sample Processing:
1. Check patient identifiers
2. Label 2 X 2ml O-ring tubes with sample ID#
3. Vortex the tube with swab for 10-15 seconds
4. Let tube stand for 5 minutes in a rack
5. Remove cap
6. Use sterile 1ml pipette to pipet 0.5ml of solution into each of 2 tubes.
7. Dispose of tube with swab and cap in biohazard waste.
8. Place the 2 X 2ml O-ring tubes in a storage box in -80°C freezer

RNA Extraction:
- RNA is extracted from stored swab samples according to QIAamp® 96 Virus QIAcube® HT Handbook.[3]

PCR Protocol:
- Extracted RNA is analyzed according to TaqPath™COVID‑19 CE‑IVD RT‑PCR Kit protocol.[4]

Processing of Blood Tubes

Equipment needed

- Sample
- 2 inch freezer storage boxes
- Centrifuge (for example: Eppendorf 5702 and Sorvall ST16R centrifuges)
- 1 X 2ml cryovial storage tube
- 2 X 10ml pipette
- 2 X 5ml pipette
- 15ml Falcon conical tube
- Serologic Pipette device
- Biohazard bag in BSC
- 11 copies of sample labels
- Blood tube racks
- 200ul pipette tips
- 200ul pipette
- Phosphate Buffered Saline (PBS) (Sigma P5493-1L)
- Water bath at 56°C
- COVID-19 antibody rapid diagnostic test kit (RDT)
- Chromatography paper - if saving dried blood spots

If PBMC collected, you need this in addition to above per sample

- 5 X cryovial
- 1 X 15ml conical tube
- 20ul pipettor tips
- 20ul pipettor
- 3X 5ml pipettes
- 1 X 10ml pipette
- PBMC Freezing medium 1 (see end for contents)
- PBMC Freezing medium 2 (see end for contents)
- Disposable cell counter slide for Nexcelom Auto 2000 (SD100)
- Mr Freezy (Thermo Fisher 5100-0001), or equivalent controlled rate freezing container
- Isopropyl alcohol
- 0.4% trypan blue stain (Invitrogen T10282)
- 0.65ml centrifuge tubes
- 1ml pipette tips

Protocol RDT (rapid diagnostic test) and (dried blood spot) DBS:

1. Confirm patient samples received in the lab
2. Place EDTA tube into sample racks

3. Open COVID RDT and apply label with sample ID#
4. Conduct RDT per manufacturer's protocol
5. While waiting for the RDT test result, spot four 50µL aliquots of whole blood onto a piece of chromatography paper labeled with the participant ID and date and time of blood collection.
    a. Let dry in the biosafety hood (can also be overnight).
    b. Once dry, place into a plastic storage bag labeled with a sample ID#
    c. Store DBS in plastic storage bag at -80°C
6. Take a photo of the RDT result with participant ID label visible using the digital camera.
    a. Save photos on the encrypted drive.
7. Once RDTs are completed, move to the next step.

Protocol Plasma Processing and PBMC Collection:
1. Use the two Ficoll tubes for this sample processing. Processing should start within 2 hours of collection.
2. Record time of the start of processing on the Sample Tracking spreadsheet
3. Centrifuge the two Ficoll tubes at 1,600 X g for 30 minutes at 22°C using the Sorvall ST16R centrifuge **(during this time, can process the nasal swab)**

Plasma Collection from Ficoll Tubes:
1. Label 2 X 2ml cryovials with sample ID# label
2. Label 1 X 15ml Falcon tube with sample ID# label
3. Remove plasma (top clear yellow layer) from Ficoll tubes into a 15ml falcon tube using a 10ml pipette. Do not disturb the hazy layer below. You may leave some plasma.
4. Confirm caps of 15ml Falcon tubes are on tight.
5. If the sample is moving on to harvest PBMC, store tubes of plasma at 4°C and move to step 1 of PBMC Collection from Ficoll Tubes (only proceed if RDT positive or a sample collected at enrollment, 0 weeks, or 12 weeks).
6. Return back to complete plasma aliquoting during the first PBS wash of PBMC Collection process.
7. Heat inactivate the plasma for 30 minutes at 56°C, mix every 5 minutes.
8. Bring 15ml Falcon tube to Eppendorf 5702 centrifuge and spin 1,500 X g for 10 minutes to pellet red blood cells and protein aggregate.
9. Bring back inside BSC.
10. Carefully transfer 1.5ml of the supernatant (plasma) using 2ml pipette into two 2-ml sterile cryovials with screw top lids with O-rings using a sterile pipette tip.
11. Cap vials and place into freezer storage boxes in the -80°C freezer.

PBMC collection:[5]

1. Label a 15ml Falcon tube with sample ID#.

2. Label 5 cryovials with sample ID#.
3. Transfer the remaining plasma and the whitish, mononuclear layer (everything above the gel layer) using a serological pipette, to a 15 ml Falcon tube, pooling the mononuclear layer from each Ficoll containing vacutainer per sample ID# into one conical tube.
4. Add 1x PBS to bring the total volume in the conical tube to 15 ml.
5. Cap tube and invert 5 times.
6. Centrifuge (with brake and acceleration OFF) 20 min, 500 x g, 22°C.
7. Return the conical tube to the hood and aspirate all but ~500 µl of the PBS without disturbing the pellet. (PBMC yield is greater if ~200 µl of PBS is left above the pellet at this stage).
8. Add fresh 1x PBS to bring the volume to 10 ml. Resuspend the pellet gently. Cap tube and invert 5 times. Centrifuge (with brake and acceleration OFF) 20 min, 500 x g, 22 °C
9. Aspirate as much supernatant/PBS as possible without disturbing the pellet. Resuspend pellet by adding in 1.5ml PBMC Freezing Medium 1.
10. Add 1.5ml PBMC Freezing Medium 2 to the cell/medium solution. Vortex gently.
11. Aliquot 10 µl of the cell solution into a 0.65 ml microcentrifuge tube. Add 10 µl of 0.4% trypan blue stain into the 0.65 ml microcentrifuge tube and mix by pipetting several times.
12. Pipette 20 µl of the mixture into a cell counting chamber slide (SD100 Cellometer) and place slide into the cell counter within 3 min of mixing. Zoom in and focus the cells. Press the "Count Cells" to obtain PBMC count.
13. *If the viable PBMC number is between 3 and 6 million live cells per milliliter (mc/ml),* aliquot 1mL into each cryovial. Store PBMCs in up to 3 cryovials at a concentration of at least 3 mc/ml each.
14. *If the viable PBMC number is above 6 million cells per milliliter,* add equal parts of Freezing Medium 1 and Freezing Medium 2 to get concentration to be between 3 and 6 mc/ml. Aliquot 1mL into each cryovial.
15. *If the viable PBMC number is below 3 mc/ml*, calculate the total number of cells using an excel spreadsheet calculator *or* by multiplying the viable mc/ml by 3 ml (which is the total volume the cells are in at this time) and by 2 (which is the trypan blue dilution factor). Determine a final volume so that the concentration is at least 3mc/mL.
    a. Centrifuge the conical tube containing the cells/freezing medium solution for 5 min at 300 x g (brake and acceleration OFF). After centrifugation, aspirate the appropriate volume of freezing medium (supernatant) so the amount remaining is the final volume calculated above.
    b. Resuspend the pellet in the remaining supernatant and aliquot at least 3 mc/ml into the appropriate number of cryovials (1-4) at 1 ml/cryovial. Final freezing medium is 10% Dimethyl Sulfoxide (DMSO)/20% Fetal Bovine Serum (FBS)/70% Roswell Park Memorial Institute (RPMI) 1640.
    c. Document the cell count per cryovial and the number the cryovials and record the cell concentration for each vial.

Transfer the cryovials to a controlled rate freezing container (Mr.Freezy, must add isopropanol to Mr Freezy before use) and put at -80ºC for at least 12 hr after which time the cryovials may be transferred to a cryobox and put in a liquid nitrogen tank (vapor phase) for long term storage.

Processing of Tasso Serum Sample

Equipment needed:

- Patient samples in a biohazard bag
- 3 X patient sample label (2 for nasal swabs, 1 for serum)
    - Label should include: Study ID#, date of processing, type of sample
- Microcentrifuge with swing bucket rotor
- Microcentrifuge tubes (0.5mL capacity, freezer stable, sterile)
- Cryo tubes (freezer stable, sterile cryovials with O-ring)

Ensure all steps are carried out using the proper biosafety designation and PPE usage per laboratory or institutional protocols.

Protocol:

1. Remove blood and swab samples from the biohazard bag inside a biosafety cabinet (BSC).
2. Label samples with patient labels (confirm that label is the same as on the sample bag).
3. Nasal swab samples:
    a. In the BSC, label cryo tubes for each participant.
    b. Aliquot 0.5 mL of sample into each cryo tube.
    c. Store in a freezer box in the -80°C freezer with other study samples.
4. Blood samples:
    a. Ensure that the swing bucket rotor is in the Eppendorf 5702 centrifuge. In the BSC, label 0.5 mL microcentrifuge tubes for each participant.
    b. Spin samples at 1500 x g for 10 minutes. If a balance is needed, use a microcentrifuge tube filled with water.
    c. In the hood, aliquot serum into a labeled 0.5 mL microcentrifuge tube.
    d. Store samples in the study freezer box in a -80°C freezer.

Enzyme-linked immunosorbent assay (ELISA) Protocol

Equipment:
ELISA plate washer
ELISA plate reader
Centrifuge to spin samples and controls
Pipette tips - P1000, P200, P10
Pipettors - P1000, P200, P20, multichannel pipettors if available
1mL dilution tubes
ELISA plates

Reagents:
.05 % Tris-buffered saline + Tween (TBST)
100 mL 10X Tris-buffered saline (TBS)
900 mL autoclaved distilled deionized (DD) water
500 uL Tween 20

.2% TBST
100 mL 10X TBS
900 mL autoclaved DD water
2000 uL Tween 20

3M NaOH
120 grams in 1L of DD water

Antigen
- Concentrated stock antigen vials should be kept at -80C.
- A stock solution of antigen should be prepared for use and storage at 4°C: use 1X TBS to dilute stock antigen to a final concentration of 4mg/mL.

Secondary Antibodies
- Goat anti-human IgG (Sigma), IgA (Abcam) and IgM (Sigma) are each diluted at 1:2500 in Blocking Buffer prior to use.

PNPP Substrate
- P-Nitrophenyl phosphate tablets (Sigma), one gold and one silver tab dissolved into DD H20.

Other Reagents:
DD water
Non-fat dry milk

Reagents Prepared Day of Run:

	1 ELISA Plate	2 ELISA Plates	3 ELISA Plates	4 ELISA Plates
**Antigen**<br>***Taken from stock solution kept at 4°C.***	5 uL antigen<br>5 mL 1XTBS	10 uL antigen<br>10 mL 1XTBS	15 uL antigen<br>15 mL 1XTBS	20 uL antigen<br>20 mL 1XTBS
**Blocking Buffer (3% Milk)**	40 mL .05% TBST<br><br>1.2 g milk	70 mL .05% TBST<br><br>2.1 g milk	100 mL .05% TBST<br><br>3 g milk	130 mL .05% TBST<br><br>3.9 g milk
**Secondary Antibody Mixture**	2 uL each of IgG, IgA, and IgM<br><br>5 mL Blocking Buffer	4 uL each of IgG, IgA, and IgM<br><br>10 mL Blocking Buffer	6 uL each of IgG, IgA, and IgM<br><br>15 mL Blocking Buffer	8 uL each of IgG, IgA, and IgM<br><br>20 mL Blocking Buffer
**PNPP Substrate**	5 mL DD Autoclaved water<br><br>1 x 5 mL PNPP Tablets	10 mL DD Autoclaved water<br><br>2 x 5mL PNPP Tablets	15 mL DD Autoclaved water<br><br>3 x 5mL PNPP Tablets	20 mL DD Autoclaved water<br><br>1 x 20 mL PNPP Tablets

Note: *When preparing secondary antibody mixture, ensure you have 5 mL of blocking buffer. When preparing PNPP mixture, ensure you have 5 mL of DD water. If the volume is less than 5mL, you will run out of the mixture prematurely.*

ELISA sample prep:

- Create an ELISA layout with each sample to be tested in duplicate.
- Samples, controls (Negative and positive controls)
  - First spin samples and controls at 840xg for 5min
  - Dilute to 1:20 in Blocking Buffer in BSC.

ELISA Plate Layout Example:

	1	2	3	4	5	6	7	8	9	10	11	12
**A**	#1	#2	#3	#4	#5	#6	#7	#8	#9	#10	#11	#12
**B**	#1	#2	#3	#4	#5	#6	#7	#8	#9	#10	#11	#12
**C**	#13	#14	#15	#16	#17	#18	#19	#20	#21	#22	#23	#24
**D**	#13	#14	#15	#16	#17	#18	#19	#20	#21	#22	#23	#24
**E**	#25	#26	#27	#28	#29	#30	#31	#32	#33	#34	#35	#36
**F**	#25	#26	#27	#28	#29	#30	#31	#32	#33	#34	#35	#36
**G**	#37	#38	#39	#40	#41	#42	Pos1	Pos1	Pos2	Pos2	Neg	Neg
**H**	#37	#38	#39	#40	#41	#42	Pos1	Pos1	Pos2	Pos2	Neg	Neg

**Pos1 - low positive control*
**Pos2 - high positive control*
**Neg - negative control*

ELISA Protocol:

1. Add 50ul of 200ng/well of your antigen diluted in 1x TBS and incubate for 1 hour at 37°C or overnight at 4°C.
    a. If stock is at 4mg/mL, dilute this by 1:1000 in 1x TBS, add 50ul to each well.
    b. 1hr incubations should be done on a rotating surface if possible
    c. For all incubations: make sure to cover ELISA plate with plastic wrap and place into a tupperware box with a damp paper towel inside to ensure even distribution of humidity.
2. Wash x3 in ELISA plate washer with 0.2% TBST, by placing directly into the washer, then blot on a dry paper towel when done.
3. Add 100 µl of Blocking Buffer and incubate for 1 hour at 37°C.
4. While blocking, make all serum dilutions in BSC according to these guidelines based on 1 ELISA Plate:
    a. For 1:20 dilution: Add 380 uL of blocking buffer into all serum dilution tubes.
    b. Add 20ul of sample into each tube. Mix 3x in the sample tube before and after adding to the dilution tube.
    c. Control volumes at 1:20:
        i. One ELISA plate: 285 uL blocking buffer + 15 uL control
        ii. Two ELISA plates: 475 uL blocking buffer + 25 uL control

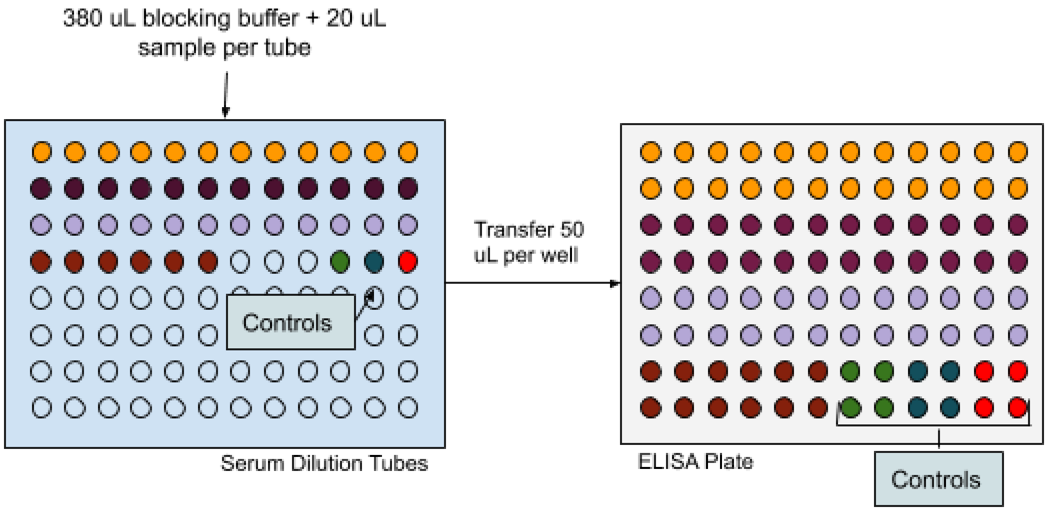

5. After blocking is completed, dump blocking buffer into sink, blot on a dry paper towel.
6. Primary Antibody: Add 50ul/well of your diluted sera or control reagents, pipette up and down 30 times before adding to ELISA to mix. Incubate at 37°C for 1 hour.
    a. While primary is incubating, make secondary antibody dilution, vortex/mix well and store at 4°C.
7. Remove your sample from the ELISA plate by carefully pipetting out in BSC into liquid waste. Do not touch the bottom of well with the pipette tip.

    a. This is done to minimize clogging due to milk waste traveling into our ELISA plate washer, if this is not a concern, can skip this step.
8. Wash x3 in ELISA plate washer with 0.2% TBST, blot on dry paper towel.
9. Secondary Antibody: Add 50 µl/well of secondary anti-human Ig (IgG=1:2500, 1gM=1:2500, IgA=1:2500) diluted in Blocking Buffer, incubate at 37°C for 1 hour.
    a. While secondary is incubating, mix PNPP substrate tablets (1:1 gold:silver tab per manufacturer protocol) with autoclaved DI H20 in dark conical tube, place on mixer/rotator at RT, do not shake to prevent bubbles/foaming.
10. While secondary is incubating, go to ELISA plate reader to log in and set up.
11. When secondary incubation is complete, flick out into sink before placing into ELISA washer (again to prevent ELISA washer clogging, can skip if this is not an issue).
12. Wash x3 in ELISA plate washer with 0.2% TBST, blot on a clean, dry paper towel.
13. Add 50ul/well of PNPP substrate- start timer at the start of first row.
14. Read ELISA plate at 405nm Absorbance every 2 minutes from 4 - 12 minutes.

Interpretation of Data Example:

ELISA plate readout (Optical Density (OD) 405nm)

	1	2	3	4	5	6	7	8	9	10	11	12
Sample	0.183	0.172	0.174	0.183	0.182	0.208	0.202	0.185	0.200	0.217	0.195	0.195
Sample Duplicate	0.196	0.158	0.171	0.180	0.189	0.213	0.215	0.177	0.196	0.198	0.178	0.190

Excel was utilized in interpreting results using the following set-up and formulas:

Average OD	0.190	0.165	0.173	0.182	0.186	0.211	0.215	0.181	0.198	0.208	0.187	0.193
P/N ratio	0.998	0.869	0.909	0.956	0.977	1.109	1.098	0.953	1.043	1.093	0.982	1.014
OD variance	3.430	4.242	0.869	0.826	1.886	1.187	3.117	2.209	1.010	4.578	4.557	1.298
								Neg Control Avg			0.190	

Calculations:

Average: (Duplicate 1 + Duplicate 2) / 2

Positive/Negative ratio (P/N ratio): Duplicate OD Average / Negative Control OD Average

OD variance*: | (Duplicate 1 - Duplicate 2 / Duplicate 1 + Duplicate 2)*100 |

Repeat those that are >25% discordant if they are both above the OD cutoff for the ELISA

References

1. Birger, R, et al. "Asymptomatic shedding of respiratory virus among an ambulatory population across seasons." mSphere 3(4) (2018).
2. Galanti, Marta, et al., "Longitudinal active sampling for respiratory viral infections across age groups" Influenza and Other Respiratory Viruses, 13(3) (2019).
3. QIAamp® 96 Virus QIAcube® HT Handbook. Qiagen, 2016 pp 23-28.: https://www.qiagen.com/us/resources/resourcedetail?id=c80685c0-4103-49ea-aa72-8989420e3018&lang=en
4. TaqPath™COVID-19 CE-IVD RT-PCR Kit protocol. Thermo Fisher Scientific. Pleasanton, California, 2020, pp. 43-48. : https://assets.thermofisher.com/TFS-Assets/LSG/manuals/MAN0019215_TaqPathCOVID-19_CE-IVD_RT-PCR%20Kit_IFU.pdf
5. Weckle A, Aiello AE, Uddin M, Galea S, Coulborn RM, Soliven R, Meier H, Wildman DE. Rapid Fractionation and Isolation of Whole Blood Components in Samples Obtained from a Community-based Setting. J Vis Exp. 2015 Nov 30;(105):52227. doi: 10.3791/52227. PMID: 26649992; PMCID: PMC4692771.
